# Approximation algorithm for rearrangement distances considering repeated genes and intergenic regions

**DOI:** 10.1186/s13015-021-00200-w

**Published:** 2021-10-13

**Authors:** Gabriel Siqueira, Alexsandro Oliveira Alexandrino, Andre Rodrigues Oliveira, Zanoni Dias

**Affiliations:** Institute of Computing, University of Campinas, Campinas, Brazil

**Keywords:** Genome rearrangement, Intergenic regions, Reversal

## Abstract

The rearrangement distance is a method to compare genomes of different species. Such distance is the number of rearrangement events necessary to transform one genome into another. Two commonly studied events are the transposition, which exchanges two consecutive blocks of the genome, and the reversal, which reverts a block of the genome. When dealing with such problems, seminal works represented genomes as sequences of genes without repetition. More realistic models started to consider gene repetition or the presence of intergenic regions, sequences of nucleotides between genes and in the extremities of the genome. This work explores the transposition and reversal events applied in a genome representation considering both gene repetition and intergenic regions. We define two problems called Minimum Common Intergenic String Partition and Reverse Minimum Common Intergenic String Partition. Using a relation with these two problems, we show a $\Theta \left( k \right)$-approximation for the Intergenic Transposition Distance, the Intergenic Reversal Distance, and the Intergenic Reversal and Transposition Distance problems, where *k* is the maximum number of copies of a gene in the genomes. Our practical experiments on simulated genomes show that the use of partitions improves the estimates for the distances.

## Introduction

In the field of Computational Biology, when analyzing the relationship between two genomes, one can estimate the evolutionary distance by calculating the number of mutations necessary to transform one genome into another. These mutations can be *non-conservative* (i.e., affect the quantity of genetic material), which is the case of insertion, deletion, duplication, or substitution of individual nucleotides [1–3], or the mutations can be *conservative* (i.e., do not insert or remove genetic material), which is the case of the *conservative genome rearrangement events* [4], which affect only the order and orientation of genes in the genome.

Some conservative events affect a single chromosome, such as the *reversal*, which inverts a sequence of genes, and the transposition, which exchanges the position of two consecutive sequences of genes. There are also events that may affect more than one chromosome, such as translocation, which swaps extremities of two chromosomes. The translocation and reversal events can be simulated by the *Double-Cut-and-Join* (DCJ) [5] operation, which cuts the genome at two positions and creates two new adjacencies by joining the four extremities affected by these cuts. This work focuses on the reversal and transposition events, consequently, we only consider genomes with a single chromosome.

When comparing genomes with a rearrangement-based distance, one must select a *r*earrangement model (i.e., the set of allowed rearrangement events) and find a representation for the genomes suitable to the selected model. With a given model, a rearrangement distance problem aims at finding the minimum number of allowed rearrangement events necessary to transform one genome into another.

Genomes can be represented by a string, where each character represents a gene. There may be multiple genes represented by the same characters, those genes constitute a gene family.

If we assume that there are no replicated characters, the characters are usually represented by integer numbers, and a string of size *n* corresponds to a permutation of numbers from 1 to *n*. In this case, when comparing two genomes ${\mathcal {G}}$ and ${\mathcal {H}}$ of size *n*, one of them is represented by the identity permutation $\iota = (1~2~\ldots ~n)$ and the other by a permutation $\pi$. Consequently, finding the rearrangement distance is equivalent to finding the minimum number of allowed rearrangement events necessary to sort the permutation $\pi$.

A string (or a permutation) may also include information regarding gene orientation, and such information is encoded as signs, $+$ or −, associated with each character. In this case, we have a signed string (or a signed permutation).

When there are replicated characters, two common approaches are adopted to transform the strings into permutations. The first selects an exemplar of each gene family [6], and the second establishes a correspondence between characters of both strings [7, 8], which allows us to discriminate between multiple copies of the same character. The second approach has the advantage of losing less information but can only be applied when such correspondence can be established. In the presence of non-conservative events, the correspondence between genes may not be possible, and a preprocessing step is required to eliminate genes present in only one of the genomes.

In biological terms, this correspondence is called an *orthologous assignment*. The distance between permutations resulting from an orthologous assignment gives us a valid upper bound for the distance between the original strings. As there are multiple possible assignments, there are some strategies to find assignments that lead to lower distances [7, 8].

Recent works [9, 10] argue that considering the size of intergenic regions (i.e., number of nucleotides between genes and in the extremities of the genome) improves the estimated distances. When the sizes of intergenic regions are taken into account, the genome representation includes a string representing the gene sequence and a sequence of integers corresponding to the size of each intergenic region.

Each combination of genome representation and rearrangement model defines a different rearrangement distance problem. Table 1 shows a summary of results from the literature, considering different rearrangement distance problems and the contributions of the present work (last three rows). For each problem, we mention whether there is a known polynomial-time algorithm or an NP-hardness proof and, in the last case, what is the best known approximation factor for that problem.

**Table 1:** Summary of results for rearrangement problems

Problem	Rearrangement model	Genome representation	Complexity	Best known approximation factor
Sorting Permutations by Transpositions	Transpositions	Permutation	NP-hard [11]	1.375 [12]
Sorting Permutations by Reversals	Reversals	Permutation	NP-hard [13]	1.375 [14]
Sorting Signed Permutations by Reversals	Reversals	Signed permutation	P [15]	–
Sorting Permutations by Reversals and Transpositions	Reversals and transpositions	Permutation	NP-hard [16]	$2.8334 + \epsilon$ [17, 18]
Sorting Signed Permutations by Reversals and Transpositions	Reversals and transpositions	Signed permutation	NP-hard [16]	2 [19]
Transposition Distance on Strings	Transpositions	String	NP-hard	$12k^a$ [20, 21]
Reversal Distance on Strings	Reversals	String	NP-hard	$16k^a$ [20]
Signed Reversal Distance on Strings	Reversals	Signed string	NP-hard [22]	$16k^a$ [7, 20]
Sorting Permutations by Intergenic Transpositions	Transpositions	Permutation and sequence of integers	NP-hard [23]	3.5 [23]
Sorting Permutations by Intergenic Reversals	Reversals	Permutation and sequence of integers	NP-hard [24]	4 [24]
Sorting Signed Permutations by Intergenic Reversals	Reversals	Signed permutation and sequence of integers	NP-hard [16]	2 [16]
Sorting Permutations by Intergenic Reversals and Transpositions	Reversals and transpositions	Permutation and sequence of integers	NP-hard [24]	4.5 [24]
Sorting Signed Permutations by Intergenic Reversals and Transpositions	Reversals and transpositions	Signed permutation and sequence of integers	NP-hard [25]	3 [25]
Intergenic Reversal Distance on Strings	Reversals	String and sequence of integers	NP-hard (Theorem 1)	$6k^{a,b}$ (Corollary 2)
Intergenic Transposition Distance on Strings	Transposition	String and sequence of integers	NP-hard (Theorem 1)	$8k^a$ (Corollary 4)
Intergenic Reversal and Transposition Distance on Strings	Reversal and transposition	String and sequence of integers	NP-hard (Theorem 1)	$9k^a$ (Corollary 5)

${}^a$Some approximations depend on *k*, which is the maximum number of copies of a character in the string.

$${}^b$$Asymptotic approximation

It is worth mentioning that, to ensure an approximation, the distance between strings takes into account the result of the string partition problems [26]. Such problems seek to split two strings into sub-strings that can be concatenated in different orders to form the original strings. The way in which the sub-strings appear in each original string defines the problem. If the sub-strings must appear in the same orientation in both original strings, we have the minimum common string partition problem. If the sub-strings can appear inverted in the original strings, we have the signed minimum common string partition problem when considering signed strings, and the reverse minimum common string partition problem when considering unsigned strings.

If there is an $\ell$-approximation for the minimum common string partition problem, then there exists a $3\ell$-approximation for the transposition distance on strings problem [21]. Similarly, if there is an $\ell$-approximation for the signed minimum common string partition problem, then there exists a $2\ell$-approximation for the reversal distance on signed strings problem [7]. The same relation can be applied to the reversal distance on strings and the reverse minimum common string partition problems [26].

The best known approximation algorithms for the partition problems have factors in $O(\log n \log ^* n)$ [27], where *n* is the size of the string, and in $\Theta (k)$ [20], where *k* is the maximum number of copies of a character in the string.

This work describes approximation algorithms for the intergenic transposition distance, intergenic reversal distance, and intergenic reversal and transposition distance problems, where the representation of the genomes takes into account both repeated genes and intergenic regions. Initially, we present some definitions and formalize the problems. Next, we generalize the minimum common string partition and the reverse minimum common string partition problems to consider intergenic regions. We also present relations between the partitions and distance problems that consider intergenic regions and describe a $\Theta (k)$-approximation algorithm for the partition problems ensuring a $\Theta (k)$-approximation for the distance problems. Finally, we performed some practical tests on simulated genomes to evaluate the improvement in the estimates for the distances caused by the partition algorithms.

## Definitions

In the following definitions we use ordered sequences of elements (*lists*). The number of elements in a list *X* is denoted by |*X*|, and an element at the *i*-th position of a list *X* is denoted by $X_i$. The list $Y = rev(X)$ is equal to the list *X* in the reverse order (i.e., $|X| = |Y|$ and $Y_i$ = $X_{|X|-i+1}, \forall 1 \le i \le |X|$). A list of characters is called a *string*.

Given a string *S*, the set $\Sigma _S$ of distinct elements of *S* is the alphabet of *S* and each element of $\Sigma _S$ is called *label*. The *occurrence* of a label $\alpha$ in a string *S* is the number of characters of *S* with label $\alpha$, and is denoted by $occ(\alpha ,S)$. The *maximum occurrence* of any character in *S* is $occ(S) = \max _{\alpha \in \Sigma _S}(occ(\alpha ,S))$. A character whose label has occurrence one is called a *singleton*, and a character whose label has occurrence at least two is called a *replica*. Two strings *S* and *P* are *balanced* if $\Sigma _S = \Sigma _P$ and $occ(\alpha ,S) = occ(\alpha ,P), \forall \alpha \in \Sigma _S$. In other words, balanced strings are formed by the same characters in possibly different orders.

When modeling genomes, we consider the intergenic regions between genes represented by their sizes. Usually, an actual genome starts and ends with intergenic regions but, to construct our representation, we include two artificial genes in the beginning and end of the genome. In this process, usually called extension or capping, we use the same pair of genes for any genome.

Formally, a *genome* ${\mathcal {G}}= (g_1,\breve{g}_1,g_2,\ldots ,\breve{g}_{n-1},g_n)$ with size *n* is an interleaved sequence of *n* genes ($g_1,\ldots ,g_n$) and $n-1$ intergenic regions ($\breve{g}_1,\ldots ,\breve{g}_{n-1}$). We represent a genome ${\mathcal {G}}= (S,\breve{S})$ with a string *S* and a list of integers $\breve{S}$, such that:

- The gene $g_i$ is represented by the character $S_i$ of *S*, for $1 \le i \le n$.
- The intergenic region $\breve{g}_i$ is represented by the integer $\breve{S}_i$ of $\breve{S}$, for $1 \le i \le n-1$.

Two genomes ${\mathcal {G}}= (S,\breve{S})$ and ${\mathcal {H}}= (P,\breve{P})$ are called *co-tailed* if they have the same initial and final gene (i.e., $S_1 = P_1$ and $S_n = P_n$). Note that, any two genomes resulting from an extension are co-tailed.

**Fig. 1:**
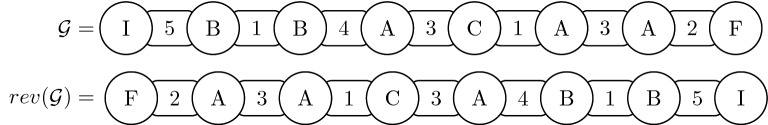
A genome ${\mathcal {G}}= (S,\breve{S})$, with $S = [I\,\, B\,\, B\,\, A\,\, C\,\, A\,\, A\,\, F]$ and $\breve{S} = [5\,\, 1\,\, 4\,\, 3\,\, 1\,\, 3\,\, 2]$, and its reverse $rev({\mathcal {G}})$. The two new genes included in the extension process are represented by the characters *I* and *F*

The *reverse* of a genome ${\mathcal {G}}= (S,\breve{S})$, denoted by $rev({\mathcal {G}})$, is a genome represented by the lists *rev*(*S*) and $rev(\breve{S})$. We say that two genomes ${\mathcal {G}}$ and ${\mathcal {H}}$ are *equal* (${\mathcal {G}}= {\mathcal {H}}$) if their correspondent strings and their correspondent integer lists are equal. Additionally, we say that two genomes ${\mathcal {G}}$ and ${\mathcal {H}}$ are *congruent* (${\mathcal {G}}\cong {\mathcal {H}}$) if ${\mathcal {G}}= {\mathcal {H}}$ or ${\mathcal {G}}= rev({\mathcal {H}})$. Figure 1 shows an example of a genome and its reverse.

Given a genome ${\mathcal {G}}= (S,\breve{S})$, the *subgenome* ${\mathcal {G}}^{i,j} = (S^{i,j}, \breve{S}^{i,j})$ is the portion of genome ${\mathcal {G}}$ between the genes $g_i$ and $g_j$. Consequently, the subgenome ${\mathcal {G}}^{i,j}$ is represented by lists $S^{i,j}$ and $\breve{S}^{i,j}$, such that:

$$\eqalign{& S_k^{i,j} = {S_{i + k - 1}},\forall 1 \leqslant k \leqslant j - 1 + 1 \cr & \breve{S} _k^{i,j} = {\breve{S}_{i + k - 1}},\forall 1 \leqslant k \leqslant j - 1 \cr}$$

A genome ${\mathcal {G}}$ contains another genome ${\mathcal {H}}$ if ${\mathcal {H}}$ is equal to some subgenome of ${\mathcal {G}}$. We denote that relation by ${\mathcal {H}}\subset {\mathcal {G}}$. We also use ${\mathcal {H}}\not \subset {\mathcal {G}}$ to indicate that ${\mathcal {G}}$ does not contain ${\mathcal {H}}$.

**Fig. 2:**
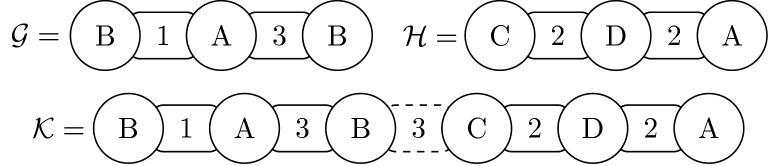
The genomes ${\mathcal {G}}= ([B\,\, A\,\, B],[1\,\, 3])$ and ${\mathcal {H}}= ([C\,\, D\,\, A],[2\,\, 2])$ combined to form the genome ${\mathcal {K}}= ([B\,\, A\,\, B\,\, C\,\, D\,\, A],[1\,\, 3\,\, 3\,\, 2\,\, 2])$. Note that, an intergenic region with size 3 was created during the combination. Besides, the genome ${\mathcal {K}}$ contains the genomes ${\mathcal {G}}$ (${\mathcal {G}}= {\mathcal {K}}^{1,3}$) and ${\mathcal {H}}$ (${\mathcal {H}}= {\mathcal {K}}^{4,6}$)

Let us define an operation of a combination of genomes (exemplified in Fig. 2). We say that a genome ${\mathcal {K}}= (Q,\breve{Q})$ is a *combination* of two genomes ${\mathcal {G}}= (S,\breve{S})$ and ${\mathcal {H}}= (P,\breve{P})$ if:

- *Q* is the concatenation of the strings *S* and *P*.
- $\breve{Q}$ is formed by the list $\breve{S}$ followed by an integer (representing the size of the intergenic region between the two genomes) and then followed by the list $\breve{P}$.

Two genomes ${\mathcal {G}}= (S,\breve{S})$ and ${\mathcal {H}}= (P,\breve{P})$ of size *n* are *balanced* if:

- The strings *S* and *P* are balanced.
- The sum of the integers correspondent to intergenic regions are the same, i.e., $\sum _{i=1}^{n} \breve{S}_i = \sum _{i=1}^{n} \breve{P}_i$

**Fig. 3:**
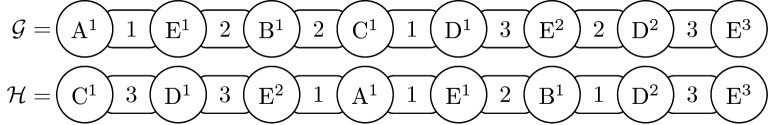
One of the possible orthologous assignments between two balanced genomes ${\mathcal {G}}=([A\,\, E\,\, B\,\, C\,\, D\,\, E\,\, D\,\, E],[1\,\, 2\,\, 2\,\, 1\,\, 3\,\, 2\,\, 3])$ and ${\mathcal {H}}=([C\,\, D\,\, E\,\, A\,\, E\,\, B\,\, D\,\, E],[3\,\, 3\,\, 1\,\, 1\,\, 2\,\, 1\,\, 3])$. The superscripts on each gene represent the assignment (characters with same label and same index are associated with each other, i.e., $\xi (X^i) = X^i$)

Given two balanced genomes ${\mathcal {G}}= (S,\breve{S})$ and ${\mathcal {H}}= (P,\breve{P})$, an *orthologous assignment* $\xi$ between them is a mapping between genes, i.e., for each gene $S_i$ of *S* there is a correspondent gene $\xi (S_i)$ in *P*. We denote the intergenic region after the gene $\xi (S_i)$ by $\xi (\breve{S}_i)$. Each singleton from *S* is associated with the singleton of same label from *P*. Each replica from *S* must be associated with a replica of same label from *P*. Note that there are multiple ways to perform the association for a replica. Figure 3 shows an orthologous assignment between two genomes ${\mathcal {G}}$ and ${\mathcal {H}}$.

**Fig. 4:**
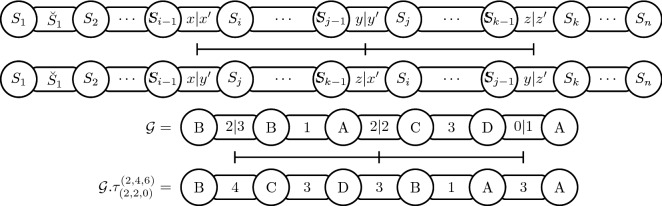
A generic representation of an intergenic transposition followed by the application of the intergenic transposition $\tau ^{\small (2,4,6)}_{\small (2,2,0)}$ on the genome ${\mathcal {G}}= ([B\,\, B\,\, A\,\, C\,\, D\,\, A],[5\,\, 1\,\, 4\,\, 3\,\, 1])$ resulting in the genome ${\mathcal {G}}.\tau ^{\small (2,4,6)}_{\small (2,2,0)} = ([B\,\, C\,\, D\,\, B\,\, A\,\, A], [4\,\, 3\,\, 3\,\, 1\,\, 3])$

Consider a genome ${\mathcal {G}}= (S,\breve{S})$ of size *n* and the numbers *i*, *j*, *k*, *x*, *y*, *z*, with $2 \le i< j < k \le n$, $0 \le x \le \breve{S}_{i-1}$, $0 \le y \le \breve{S}_{j-1}$, and $0 \le z \le \breve{S}_{k-1}$. The *intergenic transposition* $\tau ^{\small (i,j,k)}_{\small (x,y,z)}$ is an operation that transforms ${\mathcal {G}}$ into a genome ${\mathcal {G}}. \tau ^{\small (i,j,k)}_{\small (x,y,z)} = (S',\breve{S}')$, where:

$$\begin{aligned} S'&= [{{S_1\ldots S_{i-1}} \,\underline{S_j \ldots S_{k-1}}\,\underline{S_i \ldots S_{j-1}} \,{S_k \ldots S_n}}]\\ \breve{S}'&=[{{\breve{S}_1 \ldots \breve{S}_{i-2}} \,\mathop {{x+y'}}\limits _{----} \,\underline{\breve{S}_{j} \ldots \breve{S}_{k-2}} \,\mathop {{z+x'}}\limits _{----}}\\&\underline{\breve{S}_{i} \ldots S_{j-2}}\,\mathop {y+z'}\limits _{----}\, {S_{k} \ldots S_{n-1}}], \end{aligned}$$

with $x' = \breve{S}_{i-1} - x$, $y' = \breve{S}_{j-1} - y$, and $z' = \breve{S}_{k-1} - z$. Figure 4 shows a generic intergenic transposition and an example of an intergenic transposition applied in a genome ${\mathcal {G}}$.

**Fig. 5:**
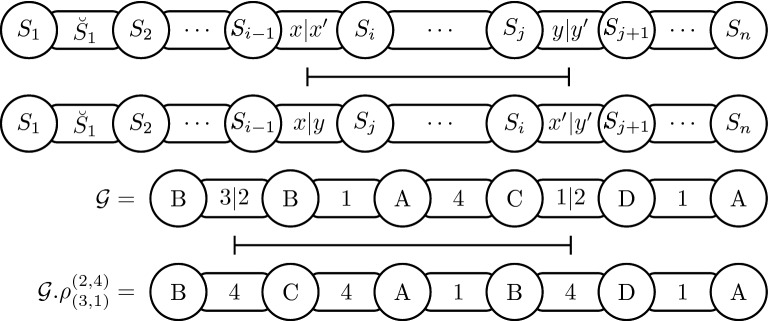
A generic representation of an intergenic reversal followed by the application of the intergenic reversal $\rho ^{\small (2,4)}_{\small (3,1)}$ on the genome ${\mathcal {G}}= ([B\,\, B\,\, A\,\, C\,\, D\,\, A],[5\,\, 1\,\, 4\,\, 3\,\, 1])$ resulting in the genome ${\mathcal {G}}.\rho ^{\small (2,4)}_{\small (3,1)} = ([B\,\, C\,\, A\,\, B\,\, D\,\, A], [4\,\, 4\,\, 1\,\, 4\,\, 1])$

Consider a genome ${\mathcal {G}}= (S,\breve{S})$ of size *n* and the numbers *i*, *j*, *x*, *y*, with $2 \le i < j \le n-1$, $0 \le x \le \breve{S}_{i-1}$, and $0 \le y \le \breve{S}_j$. The *intergenic reversal* $\rho ^{\small (i,j)}_{\small (x,y)}$ is an operation that transforms ${\mathcal {G}}$ into a genome ${\mathcal {G}}. \rho ^{\small (i,j)}_{\small (x,y)} = (S',\breve{S}')$, where:

$$\begin{aligned} S'&= [S_1 \ldots S_{i-1}\,\underline{S_j \ldots S_{i}} \ldots S_n]\\ \breve{S}'&= [{\breve{S}_1 \ldots \breve{S}_{i-2}}\, \mathop {x+y}\limits _{----}\,\underline{\breve{S}_{j-1} \ldots \breve{S}_{i}}\,\mathop {x'+y'}\limits _{----}\,{\breve{S}_{j+1} \ldots \breve{S}_{n-1}}], \end{aligned}$$

with $x' = \breve{S}_{i-1} - x$ and $y' = \breve{S}_{j} - y$. Figure 5 shows a generic reversal and an example of a reversal applied in a genome ${\mathcal {G}}$.

As shown in the following problem statements, we are interested in finding the minimum number of intergenic operations necessary to transform one genome into another. We assume that the genomes come from the extension process and, consequently, they are co-tailed.

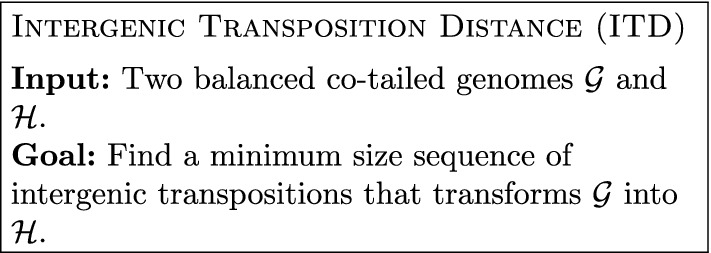

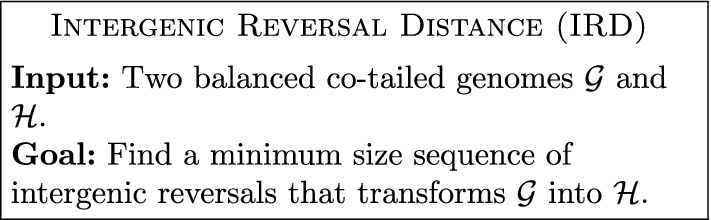

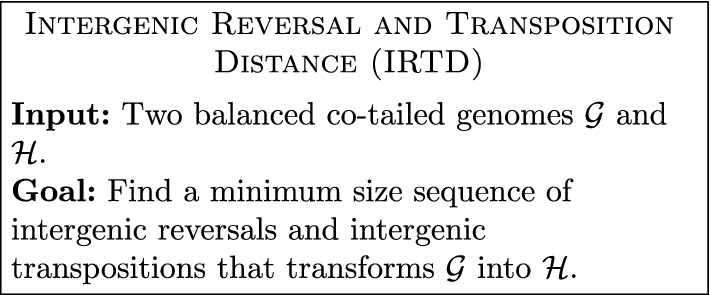







### Theorem 1

*The ITR, IRD and IRTD problems belong to the NP-hard class.*

### Proof

Directly from the fact that the correspondent problems on permutations are in the NP-hard class [23, 24]. $\square$

The minimum number of intergenic transpositions necessary to transform one genome ${\mathcal {G}}$ into another genome ${\mathcal {H}}$ is called the *intergenic transposition distance*, and it is denoted by $d_{{{\mathcal {IT}}}}({\mathcal {G}},{\mathcal {H}})$. Similarly, the minimum number of intergenic reversals necessary to transform one genome ${\mathcal {G}}$ into another genome ${\mathcal {H}}$ is called the *intergenic reversals distance*, and it is denoted by $d_{{{\mathcal {IR}}}}({\mathcal {G}},{\mathcal {H}})$. Also, the minimum number of operations that are either intergenic reversals or intergenic transpositions necessary to transform one genome ${\mathcal {G}}$ into another genome ${\mathcal {H}}$ is called the *intergenic reversals and transposition distance*, and it is denoted by $d_{\mathcal {IRT}}({\mathcal {G}},{\mathcal {H}})$.

## Intergenic Partition

In order to develop a solution for the ITD, IRD, and IRTD problems we studied two related problems called minimum common intergenic string partition and reverse minimum common intergenic string partition. To define those problems, we consider the following two types of intergenic partitions of two balanced genomes.

An *direct intergenic partition* between two balanced genomes ${\mathcal {G}}= (S,\breve{S})$ and ${\mathcal {H}}= (P,\breve{P})$ is a pair of genome sequences $({\mathbb {S}},{\mathbb {P}})$ such that:

1. The genomes of ${\mathbb {S}}$ when combined correspond to the genome ${\mathcal {G}}$.
2. The genomes of ${\mathbb {P}}$ when combined correspond to the genome ${\mathcal {H}}$.
3. It is possible to change the order of the genomes of ${\mathbb {S}}$ to obtain the genomes of ${\mathbb {P}}$ (i.e., there is at least one permutation $\phi$, from the numbers 1 to $|{\mathbb {S}}|$, such that ${\mathbb {P}}_i = {\mathbb {S}}_{\phi _i}$, $\forall ~{1 \le i \le |{\mathbb {S}}|}$).

A *reverse intergenic partition* between two balanced genomes ${\mathcal {G}}= (S,\breve{S})$ and ${\mathcal {H}}= (P,\breve{P})$ is a pair of genome sequences $({\mathbb {S}},{\mathbb {P}})$ such that:

1. The genomes of ${\mathbb {S}}$ when combined correspond to the genome ${\mathcal {G}}$.
2. The genomes of ${\mathbb {P}}$ when combined correspond to the genome ${\mathcal {H}}$.
3. It is possible to change the order and orientation of the genomes of ${\mathbb {S}}$ to obtain the genomes of ${\mathbb {P}}$ (i.e., there is at least one permutation $\phi$, from the numbers 1 to $|{\mathbb {S}}|$, such that ${\mathbb {P}}_i \cong {\mathbb {S}}_{\phi _i}$, $\forall ~{1 \le i \le |{\mathbb {S}}|}$).

In both intergenic partitions, the genomes correspondent to elements of ${\mathbb {S}}$ and ${\mathbb {P}}$ are called *blocks*, and are subgenomes of ${\mathcal {G}}$ and ${\mathcal {H}}$, respectively. As the blocks of ${\mathbb {S}}$ must be combined to form ${\mathcal {G}}$, the blocks must follow the order in which they appear in ${\mathcal {G}}$. Additionally, every gene must appear in some block. Some intergenic regions, on the other hand, do not appear in ${\mathbb {S}}$, those are the regions that must be included during the combination of the blocks. As these regions mark the points where the genome ${\mathcal {G}}$ is split into blocks, we call them *breakpoints* of ${\mathbb {S}}$. The breakpoints of ${\mathbb {P}}$ have a similar definition. Two breakpoints $\breve{X}_i$ and $\breve{Y}_j$ are called *equivalent* if the surrounding genes are equal, i.e., $X_{i} = Y_{i}$ and $X_{i+1} = Y_{i+1}$. Additionally, two breakpoints $\breve{X}_i$ and $\breve{Y}_j$ are called *congruent* if they have the same surrounding genes in possibly different positions, i.e., $X_{i} = Y_{i}$ and $X_{i+1} = Y_{i+1}$, or $X_{i} = Y_{i+1}$ and $X_{i+1} = Y_{i}$.

The $cost({\mathbb {S}},{\mathbb {P}})$ of an intergenic partition $({\mathbb {S}},{\mathbb {P}})$ is the number of breakpoints of ${\mathbb {S}}$. The cost can also be calculated by the number of blocks in ${\mathbb {S}}$ minus one. Note that, as a consequence of the third condition, both sequences ${\mathbb {S}}$ and ${\mathbb {P}}$ must have the same number of blocks and, consequently, the cost would be the same if we consider ${\mathbb {P}}$ instead of ${\mathbb {S}}$.

An intergenic partition is minimal if no two consecutive blocks can be combined to form an intergenic partition with smaller cost. An orthologous assignment between two genomes ${\mathcal {G}}$ and ${\mathcal {H}}$ associates genes of ${\mathcal {G}}$ with genes of ${\mathcal {H}}$ and, consequently, induces a unique minimal intergenic partition between ${\mathcal {G}}$ and ${\mathcal {H}}$.

Given a orthologous assignment $\xi$ between two balanced genomes ${\mathcal {G}}= (S,\breve{S})$ and ${\mathcal {H}}= (P,\breve{P})$, and the minimal intergenic partition $({\mathbb {S}},{\mathbb {P}})$ between ${\mathcal {G}}$ and ${\mathcal {H}}$ induced by $\xi$, we can distinguish between two types of breakpoint from ${\mathbb {S}}$. A breakpoint $\breve{S}_i$ is called *hard* if the genes $\xi (S_i)$ and $\xi (S_{i+1})$ are adjacent in *P*. A breakpoint is called *soft* if it is not hard, and a hard breakpoint is called *overcharged*, if $\breve{S}_i > \xi (\breve{S}_i)$, or *undercharged*, if $\breve{S}_i < \xi (\breve{S}_i)$. Additionally, we say that an intergenic transposition $\tau ^{\small (i,j,k)}_{\small (x,y,z)}$ applied to ${\mathcal {G}}$ removes *b* breakpoints of ${\mathbb {S}}$ if $cost({\mathbb {R}},{\mathbb {Q}}) = cost({\mathbb {S}},{\mathbb {P}}) - b$, where $({\mathbb {R}},{\mathbb {Q}})$ is the partition between $\tau ^{\small (i,j,k)}_{\small (x,y,z)}.{\mathcal {G}}$ and ${\mathcal {H}}$ induced by the assignment $\xi$.

### Example 1

**Fig. 6:**
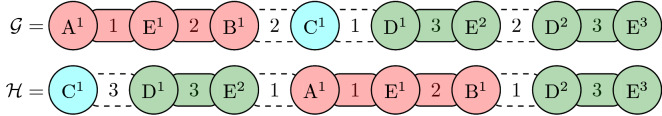
A graphical representation of the direct intergenic partition from Example 1. The intergenic regions with dashed lines are the breakpoints and each block is shown in a different color. The superscripts on each gene represent an assignment capable of inducing the partition. The breakpoint between genes $C^1$ and $D^1$ is an undercharged hard breakpoint, and the remaining breakpoints are soft

An direct intergenic partition $({\mathbb {S}}, {\mathbb {P}})$ of two genomes ${\mathcal {G}}= (S,\breve{S})$ and ${\mathcal {H}}= (P,\breve{P})$ of cost 3. Figure 6 shows a graphical representation of the partition $({\mathbb {S}},{\mathbb {P}})$ and a possible orthologous assignment capable of inducing that partition.

$$\begin{aligned} S&~=~ [A\,\, E\,\, B\,\, C\,\, D\,\, E\,\, D\,\, E]~~~~~~ \breve{S} ~=~ [1\,\, 2\,\, 2\,\, 1\,\, 3\,\, 2\,\, 3]\\ P&~=~ [C\,\, D\,\, E\,\, A\,\, E\,\, B\,\, D\,\, E]~~~~~~ \breve{P} ~=~ [3\,\, 3\,\, 1\,\, 1\,\, 2\,\, 1\,\, 3]\\ {\mathbb {S}}&~=~ [([{A\,E\,B}], {[1\,2]})\,([{C}],[{~}])\,([{D\,E}], [3])\, ([{D\,E}], [{3}])]\\ {\mathbb {P}}&~=~ [([{C}], [{~}])\,([{D\,E}], [{3}])\,([{A\,E\,B}], [{1\, 2}])\, ([{D\,E}],[{3}])] \end{aligned}$$

### Example 2

**Fig. 7:**
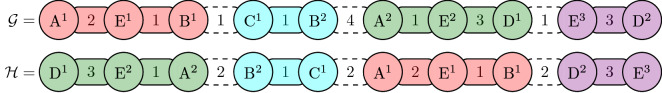
A graphical representation of the direct intergenic partition from Example 2. The intergenic regions with dashed lines are the breakpoints and each block is shown in a different color. The superscripts on each gene represent an assignment capable of inducing the partition

A reverse intergenic partition $({\mathbb {S}}, {\mathbb {P}})$ of two genomes ${\mathcal {G}}= (S,\breve{S})$ and ${\mathcal {H}}= (P,\breve{P})$ of cost 3. Figure 7 shows a graphical representation of the partition $({\mathbb {S}},{\mathbb {P}})$ and a possible orthologous assignment capable of inducing that partition.

$$\begin{aligned} S&~=~ [A\,\, E\,\, B\,\, C\,\, A\,\, E\,\, D\,\, E\,\, D]~~~~~~ \breve{S} ~=~ [2\,\, 1\,\, 2\,\, 2\,\, 1\,\, 3\,\, 2\,\, 3]\\ P&~=~ [C\,\, D\,\, E\,\, A\,\, A\,\, E\,\, B\,\, D\,\, E]~~~~~~ \breve{P} ~=~ [4\,\, 3\,\, 1\,\, 1\,\, 2\,\, 1\,\, 1\,\, 3]\\ {\mathbb {S}}&~=~ [{([{A\,E\,B}], {[2\,1]})\quad ([{C}],[{~}])\quad ([{A\,E\,D}], [{1\, 3}])}\\&~~~~~~~{([{E\,D}], [{3}])}]\\ {\mathbb {P}}&~=~ [{([{C}],[{~}])\quad ([{D\,E\,A}], [{3\,1}])\quad ([{A\,E\,B}],[{2\,1}])}\\&~~~~~~~{([{E\,D}],[3])}] \end{aligned}$$

We are interested in the minimum cost direct intergenic partition and in the minimum cost reverse intergenic partition, as shown in the following problem statements.

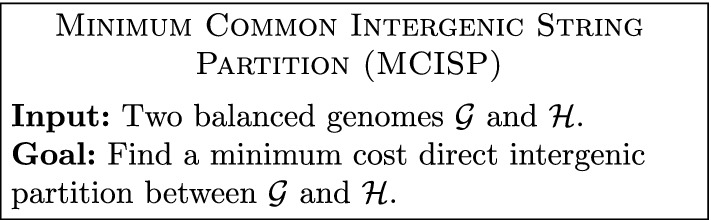

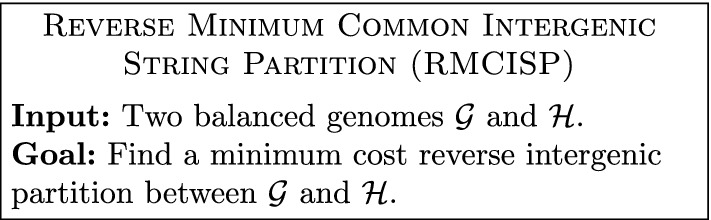





When we do not consider intergenic regions, the genomes may be represented only by the strings. In that case, there are analogous definitions for partitions.

A *direct partition* of two balanced strings *S* and *P* is a pair of string sequences $({\mathbb {S}},{\mathbb {P}})$ such that:

1. The strings of ${\mathbb {S}}$ when concatenated correspond to the string *S*.
2. The strings of ${\mathbb {P}}$ when concatenated correspond to the string *P*.
3. It is possible to change the order of the strings of ${\mathbb {S}}$ to obtain the strings of ${\mathbb {P}}$ (i.e., there is at least one permutation $\phi$, from the numbers 1 to $|{\mathbb {S}}|$, such that ${\mathbb {P}}_i = {\mathbb {S}}_{\phi _i}$, $\forall ~{1 \le i \le |{\mathbb {S}}|}$).

A *reverse partition* of two balanced strings *S* and *P* is a pair of string sequences $({\mathbb {S}},{\mathbb {P}})$ such that:

1. The strings of ${\mathbb {S}}$ when concatenated correspond to the string *S*.
2. The strings of ${\mathbb {P}}$ when concatenated correspond to the string *P*.
3. It is possible to change the order and orientation of the strings of ${\mathbb {S}}$ to obtain the strings of ${\mathbb {P}}$ (i.e., there is at least one permutation $\phi$, from the numbers 1 to $|{\mathbb {S}}|$, such that ${\mathbb {P}}_i = {\mathbb {S}}_{\phi _i}$ or ${\mathbb {P}}_i = rev({\mathbb {S}}_{\phi _i})$, $\forall ~{1 \le i \le |{\mathbb {S}}|}$).

In both cases, the cost of a partition is $|{\mathbb {S}}| - 1$ and there are problems focused on minimizing that cost.

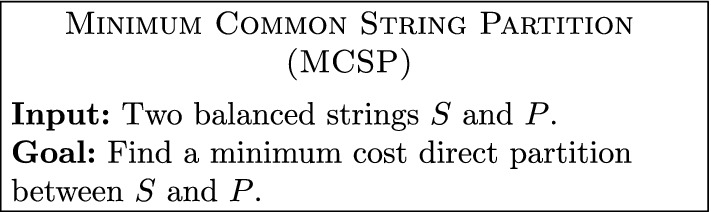

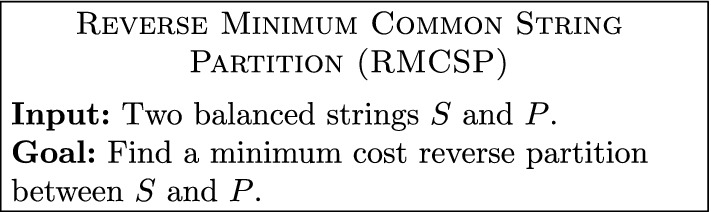





The MCSP and RMCSP problems belong to the NP-hard class [28].

### Theorem 2

  The MCISP problem belongs to the NP-hard class.

### Proof

Given an integer *p*, the decision version of the problems MCSP and MCISP aim at finding a direct partition and direct intergenic partition, respectively, of cost *p*. Considering the decision versions, let us reduce the MCSP problem to the MCISP problem.

Let the strings *S* and *P* be an instance of the MCSP problem. We construct an instance of the MCISP problem by adding the integer list $\breve{S}$ and $\breve{P}$, of size $|S|-1$, composed only by zeros. Note that, there is a partition of size *p* between *S* and *P* if and only if there is a direct intergenic partition of size *p* between $(S,\breve{S})$ and $(P,\breve{P})$. $\square$

### Theorem 3

  *The RMCISP problem belongs to the NP-hard class.*

### Proof

Analogous to the proof of Theorem 2 considering the RMCSP problem instead of MCSP. $\square$

## Correspondence between partition and distance problems

This section presents a correspondence between the partition and distance problems. Such correspondence allows us to adapt an approximation for the MCISP problem to obtain an approximation for the ITD problem, and to adapt an approximation for the RMCISP problem to obtain approximations for the IRD and IRTD problems. The following lemmas establish lower bounds for the distances based on partitions cost.

### Lemma 1

  *Let* $({\mathbb {S}},{\mathbb {P}})$ *be a minimal direct intergenic partition induced by an orthologous assignment between two balanced genomes* ${\mathcal {G}}= (S,\breve{S})$ and ${\mathcal {H}}= (P,\breve{P})$. *For any intergenic transposition* $\tau ^{\small (i,j,k)}_{\small (x,y,z)}$, *the minimal direct intergenic partition* $({\mathbb {R}},{\mathbb {Q}})$ *between the genomes* ${\mathcal {G}}.\tau ^{\small (i,j,k)}_{\small (x,y,z)}$ *and* ${\mathcal {H}}$, *induced by the same orthologous assignment, respects the restriction* $cost({\mathbb {R}},{\mathbb {Q}}) \ge cost({\mathbb {S}},{\mathbb {P}}) - 3$.

### Proof

As the direct intergenic partition $({\mathbb {R}},{\mathbb {Q}})$ must be induced by the same assignment of $({\mathbb {S}},{\mathbb {P}})$, we can only reduce the cost of the direct intergenic partition by moving the blocks to allow their combination. The intergenic transposition may be able to combine three pairs of blocks: the block ending in $S_{i-1}$ with the block starting in $S_{j}$; the block ending in $S_{k-1}$ with the block starting in $S_{i}$; and the block ending in $S_{j-1}$ with the block starting in $S_{k}$. In the best case, if all three combinations occur, we have $cost({\mathbb {R}},{\mathbb {Q}}) = cost({\mathbb {S}},{\mathbb {P}}) - 3$. $\square$

### Lemma 2

  *Let* $({\mathbb {S}},{\mathbb {P}})$ *be a minimal reverse intergenic partition induced by an orthologous assignment between two balanced genomes* ${\mathcal {G}}= (S,\breve{S})$ *and* ${\mathcal {H}}= (P,\breve{P})$. *For any intergenic transposition* $\tau ^{\small (i,j,k)}_{\small (x,y,z)}$, *the minimal reverse intergenic partition* $({\mathbb {R}},{\mathbb {Q}})$ *between the genomes* ${\mathcal {G}}.\tau ^{\small (i,j,k)}_{\small (x,y,z)}$ *and* ${\mathcal {H}}$, *induced by the same orthologous assignment, respects the restriction* $cost({\mathbb {R}},{\mathbb {Q}}) \ge cost({\mathbb {S}},{\mathbb {P}}) - 3$.

### Proof

Analogous to the proof of Lemma 1. $\square$

### Lemma 3

  *Let* $({\mathbb {S}},{\mathbb {P}})$ *be a minimal reverse intergenic partition induced by an orthologous assignment between two balanced genomes* ${\mathcal {G}}= (S,\breve{S})$ *and* ${\mathcal {H}}= (P,\breve{P})$. *For any intergenic reversal* $\rho ^{\small (i,j)}_{\small (x,y)}$, *the minimal reverse intergenic partition* $({\mathbb {R}},{\mathbb {Q}})$ *between the genomes* ${\mathcal {G}}.\rho ^{\small (i,j)}_{\small (x,y)}$ *and* ${\mathcal {H}}$, *induced by the same orthologous assignment, respects the restriction* $cost({\mathbb {R}},{\mathbb {Q}}) \ge cost({\mathbb {S}},{\mathbb {P}}) - 2$.

### Proof

Similar to the proof of Lemma 1, considering that the intergenic reversal $\rho ^{(i,j)}_{(x,y)}$ can combine up to two pairs of blocks: the block ending in $S_{i-1}$ with the block ending in $S_{j}$ and the block starting in $S_{j+1}$ with the block starting in $S_{i}$. $\square$

### Lemma 4

  *Let* $({\mathbb {S}},{\mathbb {P}})$ *be a direct intergenic partition of minimum cost between two balanced genomes* ${\mathcal {G}}= (S,\breve{S})$ *and* ${\mathcal {H}}= (P,\breve{P})$. *Any sequence of intergenic transpositions that transforms* *S* *into* *P* *must have size at least* $\frac{cost({\mathbb {S}},{\mathbb {P}})}{3}$.

### Proof

Consider a sequence of *k* intergenic transpositions capable of transforming ${\mathcal {G}}$ into ${\mathcal {H}}$. Such sequence establishes an orthologous assignment between ${\mathcal {G}}$ and ${\mathcal {H}}$. The assignment is recovered by verifying, for each character of *S*, the new position in *P*, after the intergenic transpositions are applied.

Let $({\mathbb {R}},{\mathbb {Q}})$ be the minimal direct intergenic partition induced from the orthologous assignment. We know that $\frac{cost({\mathbb {R}},{\mathbb {Q}})}{3} \le k$, because each intergenic transposition can remove at most 3 breakpoints (Lemma 1) and *k* intergenic transpositions are sufficient to turn ${\mathbb {R}}$ into ${\mathbb {Q}}$ (i.e., *k* intergenic transpositions can remove all breakpoints). As $({\mathbb {S}},{\mathbb {P}})$ is a minimum cost direct intergenic partition, we have $\frac{|({\mathbb {S}},{\mathbb {P}})|}{3} \le \frac{|({\mathbb {R}},{\mathbb {Q}})|}{3} \le k$. $\square$

### Lemma 5

  *Let* $({\mathbb {S}},{\mathbb {P}})$ *be a reverse intergenic partition of minimum cost between two balanced genomes* ${\mathcal {G}}= (S,\breve{S})$ *and* ${\mathcal {H}}= (P,\breve{P})$. *Any sequence of intergenic reversals that transforms* *S* *into* *P* *must have size at least* $\frac{cost({\mathbb {S}},{\mathbb {P}})}{2}$.

### Proof

Analogous to the proof of Lemma 4, but using Lemma 3 instead of Lemma 1. $\square$

### Lemma 6

  *Let* $({\mathbb {S}},{\mathbb {P}})$ *be a reverse intergenic partition of minimum cost between two balanced genomes* ${\mathcal {G}}= (S,\breve{S})$ *and* ${\mathcal {H}}= (P,\breve{P})$. *Any sequence composed of intergenic reversals and intergenic transpositions that transforms* *S* *into* *P* *must have size at least* $\frac{cost({\mathbb {S}},{\mathbb {P}})}{3}$.

### Proof

Analogous to the proof of Lemma 4, but using lemmas 2 and 3 instead of Lemma 1. $\square$

The next lemmas show upper bounds for the distances based on the cost of the partitions.

### Lemma 7

(Brito et al. [24]) *Let* ${\mathcal {G}}= (S,\breve{S})$ *be a genome. Given a sequence of two intergenic transpositions* $\tau ^{\small (i+1,j+1,k+1)}_{\small (\phi _i, \phi _j, \phi _k)},$ $\tau ^{\small (i+1,i+k-j+1,k+1)}_{\small (\phi '_i, \phi '_{i+k-j}, \phi '_k)}$, *applied in this order, it is possible to find values for* $\phi _i, \phi _j, \phi _k, \phi '_i,$ $\phi '_{i+k-j}, \phi '_k$ *to perform any redistribution of nucleotides within regions* $\breve{S}_i$, $\breve{S}_j$, and $\breve{S}_k$.

Note that, after the two intergenic transpositions describe in Lemma 7, the string *S* remains the same.

### Lemma 8

*Given two genomes* ${\mathcal {G}}= (S,\breve{S})$ *and* ${\mathcal {H}}= (P,\breve{P})$, *and an orthologous assignment* $\xi$ *between them. Let* $({\mathbb {S}},{\mathbb {P}})$ *be the minimal direct partition derived from the orthologous assignment* $\xi$. *If* ${\mathbb {S}}$ *has a soft breakpoint* $\breve{S}_i$ *such that* $\breve{S}_i \ge \xi (\breve{S}_i)$, *then we can apply an intergenic transposition in* ${\mathcal {G}}$ *that removes at least one breakpoint from* ${\mathbb {S}}$. *Furthermore, if* ${\mathbb {S}}$ *has at leas*t 4 *soft breakpoints and there is no breakpoint* $\breve{S}_r$, $r \ne i$, *such that* $\breve{S}_r \ge \xi (\breve{S}_r)$, *we can choose an intergenic transposition that does not create overcharged* breakpoints.

### Proof

Consider the gene $S_j$ of *S*, such that the genes $\xi (S_i)$ and $\xi (S_j)$ are adjacent in *P* and the position of $\xi (S_j)$ in *P* is greater than the position of $\xi (S_i)$. Note that $S_j \ne S_{i+1}$, otherwise this would be a hard breakpoint. Besides, note that $\breve{S}_{j-1}$ is a breakpoint.

Initially, suppose that $j > i$. Let $\breve{S}_k$ be a breakpoint such that $k < i$ or $k \ge j$. Such breakpoint must exist, otherwise $(\breve{S}_1,\ldots , \breve{S}_i)$ and $(\breve{S}_j,\ldots ,\breve{S}_{n})$ would have no breakpoints and, since $\xi (S_i)$ and $\xi (S_j)$ are adjacent and $S_j \ne S_{i+1}$, there is no valid value for $S_{i+1}$.

If $k < i$, an intergenic transposition $\tau ^{(k+1,i+1,j)}_{(x,y,z)}$ turns the pairs $(S_k, S_{i+1}),$ $(S_{j-1}, S_{k+1})$, and $(S_{i}, S_{j})$ adjacent in the new genome. Also, we can set *x*, *y*, and *z* to ensure that the intergenic region between $S_i$ and $S_j$ is not a breakpoint, since $\breve{S_i} \ge \xi (\breve{S}_i)$. Note that no breakpoints are introduced, since the affected pairs are all breakpoints. Additionally, let us assume that the region between $S_k$ and $S_{i+1}$ would become an overcharged breakpoint, that ${\mathbb {S}}$ has at least 4 breakpoints, and that there is no breakpoint $\breve{S}_r$, $r \ne i$, such that $\breve{S}_r \ge \xi (\breve{S}_r)$. In that case, let $\breve{S}_{\ell }$ be a breakpoint with $\ell \ne i$, $\ell \ne j-1$, and $\ell \ne k$. We can replace the intergenic transposition $\tau ^{(k+1,i+1,j)}_{(x,y,z)}$ to ensure that no overcharged breakpoints are added. Each case leads to an intergenic transposition choice as follows:

- If $\ell < k$, we can use the intergenic transposition $\tau ^{\small (\ell +1,i+1,j)}_{\small (x,y,z)}$ to turn the pairs $(S_{\ell }, S_{i+1})$, $(S_{j-1},S_{\ell +1})$, and $(S_{i},S_{j})$ adjacent in the new genome. Note that the region between $S_{\ell }$ and $S_{i+1}$ is not a hard breakpoint, because $S_{k}$ already comes before $S_{i+1}$ in *P*.
- If $\ell \ge j$, we can use the intergenic transposition $\tau ^{\small (i+1,j,\ell +1)}_{\small (x,y,z)}$ to turn the pairs $(S_{i}, S_{j})$, $(S_{\ell },S_{i+1})$, and $(S_{j-1},S_{\ell +1})$ adjacent in the new genome.
- If $\ell > k$ and $\ell < i$, we can use the intergenic transposition $\tau ^{\small (\ell +1,i+1,j)}_{\small (x,y,z)}$ to turn the pairs $(S_{\ell }, S_{i+1})$, $(S_{j-1},S_{\ell +1})$, and $(S_{i},S_{j})$ adjacent in the new genome.
- If $\ell > i$ and $\ell < j - 1$, we can use the intergenic transposition $\tau ^{\small (k+1,i+1,\ell +1)}_{\small (x,y,z)}$ to turn the pairs $(S_{k}, S_{i+1})$, $(S_{\ell },S_{k+1})$, and $(S_{i},S_{\ell +1})$ adjacent in the new genome. In that case, we do not have $(S_{i}, S_{j})$, but we can set *x*, *y*, and *z* to ensure that the intergenic region between $S_{k}$ and $S_{i+1}$ is not a breakpoint. We also ensure that the region between $S_{i}$ and $S_{\ell +1}$ is not a hard breakpoint, because $S_{j}$ already comes after $S_{i}$ in *P*.

Note that, if the region between $S_{j-1}$ and $S_{k+1}$, $S_{j-1}$ and $S_{\ell +1}$, or $S_{\ell }$ and $S_{k+1}$ becomes a hard breakpoint, we can choose the values of *x*, *y*, and *z* to ensure that it becomes an undercharged breakpoint.

If $k \ge j$, an intergenic transposition $\tau ^{\small (i+1, j, k+1)}_{\small (x,y,z)}$ turns the pairs $(S_i, S_j)$, $(S_k, S_{i+1})$, and $(S_{j-1}, S_{k+1})$ adjacent in the new genome. Also, we can set *x*, *y*,  and *z* to ensure that the intergenic region between $S_i$ and $S_j$ is not a breakpoint, since $\breve{S_i} \ge \xi (\breve{S}_i)$. Additionally, if ${\mathbb {S}}$ has at least 4 breakpoints and there is no breakpoint $\breve{S}_r$, $r \ne i$, such that $\breve{S}_r \ge \xi (\breve{S}_r)$, we may replace the intergenic transposition, as in the previous case, to ensure that it does not create overcharged breakpoints.

Now, suppose that $i > j$. Let $\breve{S}_k$ be a breakpoint such that $k < i$ and $k \ge j$. Such breakpoint must exist, otherwise $(\breve{S}_j,\ldots , \breve{S}_i)$ would have no breakpoints, which is a contradiction because the position of $\xi (S_j)$ in *P* is greater than the position of $\xi (S_i)$. An intergenic transposition $\tau ^{\small (j,k+1,i+1)}_{\small (x,y,z)}$ turns the pairs $(S_{j-1}, S_{k+1}),$ $(S_{i}, S_{j})$, and $(S_{k}, S_{i+1})$ adjacent in the new genome. Also, we can set *x*, *y*,  and *z* to ensure that the intergenic region between $S_i$ and $S_j$ is not a breakpoint, since $\breve{S_i} \ge \xi (\breve{S}_i)$. Additionally, if ${\mathbb {S}}$ has at least 4 breakpoints and there is no breakpoint $\breve{S}_r$, $r \ne i$, such that $\breve{S}_r \ge \xi (\breve{S}_r)$, we may replace the intergenic transposition, as in the previous case, to ensure that it does not create overcharged breakpoints. $\square$

### Lemma 9

*Given two genomes* ${\mathcal {G}}= (S,\breve{S})$ *and* ${\mathcal {H}}= (P,\breve{P})$, *and an orthologous assignment* $\xi$ *between them, it is possible to turn* ${\mathcal {G}}$ *into* ${\mathcal {H}}$ *using at most* $cost({\mathbb {S}},{\mathbb {P}}) + 1$ *intergenic transpositions, where* $({\mathbb {S}},{\mathbb {P}})$ *is the minimal direct partition derived from the orthologous assignment* $\xi$.

### Proof

We will describe how to apply at most $cost({\mathbb {S}},{\mathbb {P}}) + 1$ intergenic transpositions in ${\mathcal {G}}$ to remove all breakpoints from ${\mathbb {S}}$ and, consequently, to turn ${\mathcal {G}}$ into ${\mathcal {H}}$. The intergenic transpositions are applied according to the following cases:

1. If there are two or more overcharged breakpoints in ${\mathbb {S}}$: Let $\breve{S}_i$ and $\breve{S}_j$ be two overcharged breakpoints and let $\breve{S}_k$ be another breakpoint in ${\mathbb {S}}$ (such breakpoint must exist since there are overcharged breakpoints). We can use two intergenic transpositions (Lemma 7) to move the exceeding nucleotides from $\breve{S}_i$ and $\breve{S}_j$ to the intergenic region $\breve{S}_k$.
2. If there exists a soft breakpoint $\breve{S}_i$ in ${\mathbb {S}}$ such that $\breve{S}_i \ge \xi (\breve{S}_i)$: We can use one intergenic transposition (Lemma 8) to remove at least one breakpoint from ${\mathbb {S}}$. Note that if there is no overcharged breakpoint this case must occur, otherwise the amount of intergenic region in $\breve{S}$ would be greater than the amount of intergenic region in $\breve{P}$, which is not possible.
3. If there exists only one overcharged breakpoint $\breve{S}_j$ in ${\mathbb {S}}$ and there exists no soft breakpoint $\breve{S}_i$ in ${\mathbb {S}}$ such that $\breve{S}_i \ge \xi (\breve{S}_i)$: In that case $\breve{S}_j$ must have $\xi (\breve{S}_j) + \sum _{b \in B} \xi (b) - b$ nucleotides, where *B* is the set of breakpoints distinct from $\breve{S}_j$, otherwise the amount of intergenic region in $\breve{S}$ would be different from the amount of intergenic region in $\breve{P}$. We consider two sub-cases:
  - If there is an undercharged breakpoint $\breve{S}_k$: From the quantity of nucleotides on $\breve{S}_j$, we have $\breve{S}_j + \breve{S}_k \ge \xi (\breve{S}_j) + \xi (\breve{S}_k)$. If there exists another breakpoint $\breve{S}_\ell$, then we can use two intergenic transpositions (Lemma  7) to move the necessary number of nucleotides from $\breve{S}_j$ to $\breve{S}_k$ and the exceeding number of nucleotides from $\breve{S}_j$ to $\breve{S}_\ell$. Otherwise, since these are the only breakpoints, we have $\breve{S}_j + \breve{S}_k = \xi (\breve{S}_j) + \xi (\breve{S}_k)$. We can use two intergenic transpositions to redistribute the number of nucleotides between these two regions and remove these two breakpoints as well.
  - If there is no undercharged breakpoint: There exist at least 3 soft breakpoints, because there must exist a soft breakpoint to ensure the correct quantity of nucleotides and there is no direct intergenic partition with only 1 or 2 soft breakpoints. In that case, we can use two intergenic transpositions (Lemma  7) to move the exceeding number of nucleotides from $\breve{S}_j$ to a soft breakpoint. Afterwards, we can apply intergenic transpositions from Lemma 8 to remove all soft breakpoints and ensure that no overcharged breakpoint is inserted while there are at least 4 breakpoints. When there are 3 breakpoints, at least one will be removed and the others will become hard breakpoints. As there are no longer soft breakpoints the remaining breakpoints will be removed by cases 1 and 3(*a*).

With one exception, we remove at least one breakpoint per intergenic transposition. In this way, we can transform ${\mathcal {G}}= (S, \breve{S})$ into ${\mathcal {H}}= (P, \breve{P})$ using at most $cost({\mathbb {S}},{\mathbb {P}}) + 1$ intergenic transpositions. $\square$

### Lemma 10

(Brito et al. [24]) *Given two genomes* ${\mathcal {G}}= (S,\breve{S})$ *and* ${\mathcal {H}}= (P,\breve{P})$, *and an orthologous assignment* $\xi$ *between them, it is possible to turn* ${\mathcal {G}}$ *into* ${\mathcal {H}}$ *using at most* $2 cost({\mathbb {S}},{\mathbb {P}})$ intergenic *reversals, where* $({\mathbb {S}},{\mathbb {P}})$ *is the minimal reverse partition derived from the orthologous assignment* $\xi$.

### Lemma 11

(Brito et al. [24]) *Given two genomes* ${\mathcal {G}}= (S,\breve{S})$ *and* ${\mathcal {H}}= (P,\breve{P})$, *and an orthologous assignment* $\xi$ *between them, it is possible to turn* ${\mathcal {G}}$ *into* ${\mathcal {H}}$ *using at most* $\frac{3}{2} cost({\mathbb {S}},{\mathbb {P}})$ *intergenic reversals or intergenic transpositions, where* $({\mathbb {S}},{\mathbb {P}})$ *is the minimal reverse partition derived from the orthologous assignment* $\xi$.

With the bounds presented on the previous lemmas, we can establish a relation between partition and distance problems.

### Theorem 4

  *An* $\ell$-*approximation for the MCISP problem ensures an asymptotic* $3\ell$-*approximation for the ITD problem.*

### Proof

Let ${\mathcal {G}}= (S,\breve{S})$ and ${\mathcal {H}}= (P,\breve{P})$ be two co-tailed genomes and let *p* be the size of the minimum direct intergenic partition between ${\mathcal {G}}$ and ${\mathcal {H}}$. An algorithm for the MCISP problem with approximation factor $\ell$ returns a direct intergenic partition $({\mathbb {S}},{\mathbb {P}})$, such that $p \le cost({\mathbb {S}},{\mathbb {P}}) \le \ell p$.

By Lemma 9, it is always possible to transform ${\mathcal {G}}$ into ${\mathcal {H}}$ with *k* intergenic transpositions, such that $k \le cost({\mathbb {S}},{\mathbb {P}}) + 1$. Additionally, by Lemma 4, we know that $d_{{{\mathcal {IT}}}}({\mathcal {G}},{\mathcal {H}}) \ge \frac{p}{3}$. Consequently, we have $d_{{{\mathcal {IT}}}}({\mathcal {G}},{\mathcal {H}}) \le k \le 3 \ell d_{{{\mathcal {IT}}}}({\mathcal {G}},{\mathcal {H}}) + 1$. $\square$

As a consequence of lemmas 4 and 9, we have an asymptotic 3-approximation for the intergenic transposition distance when there are no repeated genes. The best approximation factor known in the literature for that problem is 3.5 [23].

### Theorem 5

  *An* $\ell$-*approximation for the RMCISP problem ensures a* $4\ell$-*approximation for the IRD problem.*

### Proof

Let ${\mathcal {G}}= (S,\breve{S})$ and ${\mathcal {H}}= (P,\breve{P})$ be two co-tailed genomes and let *p* be the size of the minimum reverse intergenic partition between ${\mathcal {G}}$ and ${\mathcal {H}}$. An algorithm for the RMCISP problem with approximation factor $\ell$ returns a reverse intergenic partition $({\mathbb {S}},{\mathbb {P}})$, such that $p \le cost({\mathbb {S}},{\mathbb {P}}) \le \ell p$.

By Lemma 10, it is always possible to transform ${\mathcal {G}}$ into ${\mathcal {H}}$ with *k* intergenic reversals, such that $k \le 2cost({\mathbb {S}},{\mathbb {P}})$. Additionally, by Lemma 5, we know that $d_{{{\mathcal {IR}}}}({\mathcal {G}},{\mathcal {H}}) \ge \frac{p}{2}$. Consequently, we have $d_{{{\mathcal {IR}}}}({\mathcal {G}},{\mathcal {H}}) \le k \le 4 \ell d_{{{\mathcal {IR}}}}({\mathcal {G}},{\mathcal {H}})$. $\square$

### Theorem 6

  *An* $\ell$-*approximation for the RMCISP problem ensures a* $4.5\ell$-*approximation for the IRTD problem.*

### Proof

Let ${\mathcal {G}}= (S,\breve{S})$ and ${\mathcal {H}}= (P,\breve{P})$ be two co-tailed genomes and let *p* be the size of the minimum reverse intergenic partition between ${\mathcal {G}}$ and ${\mathcal {H}}$. An algorithm for the RMCISP problem with approximation factor $\ell$ returns a reverse intergenic partition $({\mathbb {S}},{\mathbb {P}})$, such that $p \le cost({\mathbb {S}},{\mathbb {P}}) \le \ell p$.

By Lemma 11, it is always possible to transform ${\mathcal {G}}$ into ${\mathcal {H}}$ with *k* intergenic reversals or intergenic transpositions, such that $k \le \frac{3}{2} cost({\mathbb {S}},{\mathbb {P}})$. Additionally, by Lemma 6, we know that $d_{\mathcal {IRT}}({\mathcal {G}},{\mathcal {H}}) \ge \frac{p}{3}$. So, we have $d_{\mathcal {IRT}}({\mathcal {G}},{\mathcal {H}}) \le k \le 4.5 \ell d_{\mathcal {IRT}}({\mathcal {G}},{\mathcal {H}})$. $\square$

## 2k-approximation for MCISP

This section presents an algorithm for the MCISP problem between two genomes ${\mathcal {G}}= (S,\breve{S})$ and ${\mathcal {H}}= (P,\breve{P})$ with an approximation factor of 2*k*, where $k = occ(S)$. The algorithm was partially inspired by the Kolman and Waleń algorithm [20] that does not consider intergenic regions.

In order to describe the algorithm we need two functions:

- $\texttt {subgen}({\mathcal {G}},{\mathcal {X}})$: the number of subgenomes of ${\mathcal {G}}$ equal to ${\mathcal {X}}$ (each of these subgenomes is an occurrence of ${\mathcal {X}}$).
- $\texttt {weight}({\mathcal {G}},{\mathcal {H}},{\mathcal {X}}) = \texttt {subgen}({\mathcal {G}},{\mathcal {X}}) - \texttt {subgen}({\mathcal {H}},{\mathcal {X}})$: a value indicating how many occurrences of ${\mathcal {X}}$ are in excess in ${\mathcal {G}}$ or in ${\mathcal {H}}$. If the value is positive ${\mathcal {G}}$ has more occurrences of ${\mathcal {X}}$ than ${\mathcal {H}}$. If the value is negative ${\mathcal {H}}$ has more occurrences of ${\mathcal {X}}$ than ${\mathcal {G}}$.

The function $\texttt {weight}$ can be generalized to work on two sequences ${\mathbb {S}}$ and ${\mathbb {P}}$ of genomes:

$$\begin{aligned} \texttt {weight}({\mathbb {S}},{\mathbb {P}},{\mathcal {X}}) = \sum _{i = 1}^{|{\mathbb {S}}|} \texttt {subgen}({\mathbb {S}}_i,{\mathcal {X}}) - \sum _{i = 1}^{|{\mathbb {P}}|} \texttt {subgen}({\mathbb {P}}_i,{\mathcal {X}}) \end{aligned}$$

### Lemma 12

  *Given two genomes* ${\mathcal {G}}= (S,\breve{S})$, ${\mathcal {H}}= (P,\breve{P})$, *and a pair* $({\mathbb {S}}, {\mathbb {P}})$ *of genome sequences, such that it satisfies the conditions* 1 *and* 2 *of direct intergenic partition, we have that* $({\mathbb {S}}, {\mathbb {P}})$ *satisfies the condition* 3 *if and only if* $\texttt {weight}({\mathbb {S}},{\mathbb {P}},{\mathcal {X}}) = 0$ *for all genomes* ${\mathcal {X}}$ *contained in* ${\mathcal {G}}$ *or in* ${\mathcal {H}}$.

### Proof

First, we argue that if the third condition is satisfied then $\texttt {weight}({\mathbb {S}},{\mathbb {P}},{\mathcal {X}}) = 0$. Assuming the third condition is satisfied, we have a permutation $\phi$, from the numbers 1 to $|{\mathbb {S}}|$, such that ${\mathbb {P}}_i = {\mathbb {S}}_{\phi _i}$, $\forall ~{1 \le i \le |{\mathbb {S}}|}$.

Let ${\mathcal {X}}$ be a genome such that ${\mathcal {X}}\subset {\mathcal {G}}$ or ${\mathcal {X}}\subset {\mathcal {H}}$. In $\texttt {weight}({\mathbb {S}},{\mathbb {P}},{\mathcal {X}})$, we are only going to count an occurrence of ${\mathcal {X}}$ in ${\mathcal {G}}$ if it is a subgenome of some block of ${\mathbb {S}}$. Similarly, we are only going to count an occurrence of ${\mathcal {X}}$ in ${\mathcal {H}}$ if it is a subgenome of some block of ${\mathbb {P}}$.

Note that, the counted occurrences of ${\mathcal {X}}$ in ${\mathcal {G}}$ are in a one-to-one correspondence with the counted occurrences of ${\mathcal {H}}$. More precisely, for a subgenome ${\mathbb {S}}_k^{i,j}$ of a block ${\mathbb {S}}_k$, such that ${\mathbb {S}}_k^{i,j} = {\mathcal {X}}$ there is a subgenome ${\mathbb {P}}_{\phi _k}^{i,j}$ of a block ${\mathbb {P}}_{\phi _k}$, such that ${\mathbb {P}}_{\phi _k}^{i,j} = {\mathcal {X}}$. Conversely, for a subgenome ${\mathbb {P}}_{\phi _k}^{i,j}$ of a block ${\mathbb {P}}_{\phi _k}$, such that ${\mathbb {P}}_{\phi _k}^{i,j} = {\mathcal {X}}$, there is a subgenome ${\mathbb {S}}_{k}^{i,j}$ of a block ${\mathbb {S}}_{k}$, such that ${\mathbb {S}}_{k}^{i,j} = {\mathcal {X}}$. Consequently, $\texttt {weight}({\mathbb {S}},{\mathbb {P}},{\mathcal {X}}) = 0$, for every genome ${\mathcal {X}}$, such that ${\mathcal {X}}\subset {\mathcal {G}}$ or ${\mathcal {X}}\subset {\mathcal {H}}$.

Now we prove that if $\texttt {weight}({\mathbb {S}},{\mathbb {P}},{\mathcal {X}}) = 0$ then the third condition is satisfied. By contradiction let us assume that there is no one-to-one correspondence between blocks of ${\mathbb {S}}$ and blocks of ${\mathbb {P}}$.

The impossibility of a correspondence may happen by four reasons: (*i*) there is a block in ${\mathbb {S}}$ that is not equal to any block of ${\mathbb {P}}$; (*ii*) there is a genome ${\mathcal {X}}$ correspondent to *r* blocks of ${\mathbb {S}}$, but $\ell < r$ blocks of ${\mathbb {P}}$; (*iii*) there is a block in ${\mathbb {P}}$ not equal to any block of ${\mathbb {S}}$; (*iv*) there is a genome ${\mathcal {X}}$ correspondent to *r* blocks of ${\mathbb {P}}$, but $\ell < r$ blocks of ${\mathbb {S}}$. Without loss of generality, we consider only the first two cases.

In case (*i*), assume that ${\mathbb {S}}_j$ is the biggest block of ${\mathbb {S}}$ not equal to any block of ${\mathbb {P}}$. As $\texttt {weight}({\mathbb {S}},{\mathbb {P}},{\mathbb {S}}_j) = 0$, we have $\sum _{i = 1}^{|{\mathbb {S}}|} \texttt {subgen}({\mathbb {S}}_i,{\mathbb {S}}_j) = \sum _{i = 1}^{|{\mathbb {P}}|} \texttt {subgen}({\mathbb {P}}_i,{\mathbb {S}}_j)$. Consequently, ${\mathbb {P}}$ must have a copy of ${\mathbb {S}}_j$ in one of its blocks. Let ${\mathbb {P}}_s$ be a block with such copy, i.e., ${\mathbb {S}}_j \subset {\mathbb {P}}_s$. If ${\mathbb {P}}_s \ne {\mathbb {S}}_j$, then ${\mathbb {S}}$ must have a copy of ${\mathbb {P}}_s$, because $\texttt {weight}({\mathbb {S}},{\mathbb {P}},{\mathbb {P}}_s) = 0$. This means that ${\mathbb {S}}$ has at least two copies of ${\mathbb {S}}_j$ and we must have another copy of ${\mathbb {S}}_j$ in ${\mathbb {P}}$. Following that argument eventually ${\mathbb {P}}$ must have a block equal to ${\mathbb {S}}_j$, contradicting the assumption of case (*i*).

In case (*ii*) we can establish a correspondence between the $\ell$ blocks of ${\mathbb {P}}$ and some of the *r* blocks of ${\mathbb {S}}$. We have at least one block of ${\mathbb {S}}$ without a correspondent in ${\mathbb {P}}$. If we ignore the blocks with correspondences when calculating the weights, the same argument of case (*i*) leads to a contradiction. $\square$

Given two genomes ${\mathcal {G}}$ and ${\mathcal {H}}$, we can easily construct a pair of genomes sequences $({\mathbb {S}}, {\mathbb {P}})$ satisfying the first two conditions of direct intergenic partition. We just have to choose which intergenic regions of ${\mathcal {G}}$ and ${\mathcal {H}}$ will be the breakpoints of ${\mathbb {S}}$ and ${\mathbb {P}}$, respectively. By Lemma 12, to ensure that $({\mathbb {S}}, {\mathbb {P}})$ is a direct intergenic partition of ${\mathcal {G}}$ and ${\mathcal {H}}$, we must choose the breakpoints such that $\texttt {weight}({\mathbb {S}},{\mathbb {P}},{\mathcal {X}}) = 0$ for all genomes ${\mathcal {X}}$ of ${\mathcal {G}}$ or ${\mathcal {H}}$.

Let ${\mathbf {T}}_{{\mathcal {G}},{\mathcal {H}}}$ be the set of all genomes ${\mathcal {X}}$, such that ${\mathcal {X}}\subset {\mathcal {G}}$ or ${\mathcal {X}}\subset {\mathcal {H}}$, and $\texttt {weight}({\mathcal {G}},{\mathcal {H}},{\mathcal {X}}) \ne 0$ and consider the subset ${{\mathbf{T}}_{{{\mathcal{G}}},{{\mathcal{H}}}}^{{{\mathbf{min}}}}} = \{ {\mathcal{X}} \in {{\mathbf{T}}_{{{\mathcal{G}}},{\mathcal{H}}}} |{\mathcal{Y}\,\not\subset \mathcal{X}},\forall {\mathcal{Y}} \in {{\mathbf{T}}_{{{\mathcal{G}}},{\mathcal{H}}}} ,{\mathcal{Y}} \ne {\mathcal{X}}\}$. Note that, to include a breakpoint in some occurrence of a genome ${\mathcal {Y}}\in {\mathbf {T}}_{{\mathcal {G}},{\mathcal {H}}} \setminus {\mathbf {T}}^{\mathbf {min}}_{{\mathcal {G}},{\mathcal {H}}}$, it suffices to include a breakpoint in the correspondent occurrence of a genome ${\mathcal {X}}\in {\mathbf {T}}^{\mathbf {min}}_{{\mathcal {G}},{\mathcal {H}}}, {\mathcal {X}}\subset {\mathcal {Y}}$. For that reason, we start by including breakpoints in elements of ${\mathbf {T}}^{\mathbf {min}}_{{\mathcal {G}},{\mathcal {H}}}$. In fact, the following lemma ensures that we must include at least one breakpoint for each element of ${\mathbf {T}}^{\mathbf {min}}_{{\mathcal {G}},{\mathcal {H}}}$.

### Lemma 13

*In order to construct a direct intergenic partition* $({\mathbb {S}}, {\mathbb {P}})$ *of two genomes* ${\mathcal {G}}$ *and* ${\mathcal {H}}$, *we must include a breakpoint in at least one copy of every element* ${\mathcal {X}}\in {\mathbf {T}}^{\mathbf {min}}_{{\mathcal {G}},{\mathcal {H}}}$.

### Proof

For a genome ${\mathcal {X}}\in {\mathbf {T}}_{{\mathcal {G}},{\mathcal {H}}}$, let $k = \texttt {weight}({\mathcal {G}},{\mathcal {H}},{\mathcal {X}})$. To ensure that $\texttt {weight}({\mathbb {S}},{\mathbb {P}},{\mathcal {X}}) = 0$, if $k > 0$ then we must include breakpoints in at least *k* copies of ${\mathcal {X}}$ in ${\mathcal {G}}$, otherwise, if $k < 0$, we must include breakpoints in at least $-k$ copies of ${\mathcal {X}}$ in ${\mathcal {H}}$. As $\texttt {weight}({\mathcal {G}},{\mathcal {H}},{\mathcal {X}}) \ne 0$, we must include at least one breakpoint in ${\mathcal {G}}$ or in ${\mathcal {H}}$, and the lemma follows. $\square$

It may be necessary to include a breakpoint in more than one occurrence of a genome ${\mathcal {X}}\in {\mathbf {T}}^{\mathbf {min}}_{{\mathcal {G}},{\mathcal {H}}}$. We define $\texttt {break}({\mathcal {X}})$ as the breakpoint associated with the genome ${\mathcal {X}}$, and when we include a breakpoint in an occurrence of ${\mathcal {X}}$ we always select a breakpoint equivalent to $\texttt {break}({\mathcal {X}})$.

To include the breakpoints, we not only must know the genomes contained in ${\mathcal {G}}$ or ${\mathcal {H}}$ with initially non-zero weight, but also keep track of genomes that acquire a non-zero weight after the inclusion of a breakpoint. For that, we generalize the sets ${\mathbf {T}}_{{\mathcal {G}},{\mathcal {H}}}$ and ${\mathbf {T}}^{\mathbf {min}}_{{\mathcal {G}},{\mathcal {H}}}$ to consider genome sequences. Given two genome sequences ${\mathbb {S}}$ and ${\mathbb {P}}$, the set ${\mathbf {T}}_{{\mathbb {S}},{\mathbb {P}}}$ comprises of genomes ${\mathcal {X}}$, such that ${\mathcal {X}}\subset {\mathbb {S}}_i$, for $1 \le i \le |{\mathbb {S}}|$, or ${\mathcal {X}}\subset {\mathbb {P}}_j$, for $1 \le j \le |{\mathbb {P}}|$, and $\texttt {weight}({\mathbb {S}},{\mathbb {P}},{\mathcal {X}}) \ne 0$. Additionally, we have the set ${\mathbf {T}}^{\mathbf {min}}_{{\mathbb {S}},{\mathbb {P}}} = \{{\mathcal {X}}\in {\mathbf {T}}_{{\mathbb {S}},{\mathbb {P}}}| {\mathcal {Y}}\not \subset {\mathcal {X}}, \forall {\mathcal {Y}}\in {\mathbf {T}}_{{\mathbb {S}},{\mathbb {P}}}, {\mathcal {Y}}\ne {\mathcal {X}}\}$.

Let us define $\texttt {break}({\mathcal {X}})$ for a genome ${\mathcal {X}}\in {\mathbf {T}}^{\mathbf {min}}_{{\mathbb {S}},{\mathbb {P}}}$. If ${\mathcal {X}}\in {\mathbf {T}}^{\mathbf {min}}_{{\mathcal {G}},{\mathcal {H}}}$, $\texttt {break}({\mathcal {X}})$ is already defined, otherwise, there must be at least one breakpoint included in some occurrence of ${\mathcal {X}}$ in ${\mathcal {G}}$ or ${\mathcal {H}}$, so $\texttt {break}({\mathcal {X}})$ is equivalent to the first breakpoint included in some occurrence of ${\mathcal {X}}$.

The algorithm that selects the breakpoints (Algorithm 1) works as follows. Initially we consider two sequences ${\mathbb {S}}^0 = [{\mathcal {G}}]$ and ${\mathbb {P}}^0 = [{\mathcal {H}}]$, each with a single block. At the *i*-th step, we produce the sequences ${\mathbb {S}}^i$ and ${\mathbb {P}}^i$ including a breakpoint in the sequences ${\mathbb {S}}^{i-1}$ and ${\mathbb {P}}^{i-1}$ based on the following rules:

- The breakpoint is included in an occurrence of a genome ${\mathcal {X}}\in \mathbf {T}^{\mathbf {min}}_{{\mathbb {S}}^{\mathbf {i-1}},{\mathbb {P}}^{\mathbf {i-1}}}$.
- If $\texttt {weight}({\mathbb {S}}^{i-1},{\mathbb {P}}^{i-1},{\mathcal {X}}) > 0$, the selected occurrence of ${\mathcal {X}}$ must come from ${\mathcal {G}}$.
- If $\texttt {weight}({\mathbb {S}}^{i-1},{\mathbb {P}}^{i-1},{\mathcal {X}}) < 0$, the selected occurrence of ${\mathcal {X}}$ must come from ${\mathcal {H}}$.
- The selected breakpoint must be equivalent to $\texttt {break}({\mathcal {X}})$.

The algorithm continues until $\texttt {weight}({\mathbb {S}}^{i},{\mathbb {P}}^{i},{\mathcal {X}}) = 0$ for all genomes ${\mathcal {X}}$ of ${\mathcal {G}}$ or ${\mathcal {H}}$, i.e., until $({\mathbb {S}}^{i},{\mathbb {P}}^{i})$ becomes a direct intergenic partition.

Let us briefly discuss the time complexity of Algorithm 1. Let *n* be the size of the input strings. First, we consider the complexity to build ${\mathbf {T}}^{\mathbf {min}}_{{\mathcal {G}},{\mathcal {H}}}$. Using the suffix tree data structure [29] (constructed in time *O*(*n*)), $\texttt {subgen}({\mathcal {G}},{\mathcal {X}})$ is computed in *O*(*n*) time, and, consequently, so is $\texttt {weight}({\mathcal {G}},{\mathcal {H}},{\mathcal {X}})$. Similarly, for a genome ${\mathcal {Y}}$, we can recover the genomes ${\mathcal {X}}$ contained in ${\mathcal {G}}$ or ${\mathcal {H}}$, such that ${\mathcal {Y}}\subset {\mathcal {X}}$, in *O*(*n*) time. Since there are $2n^2$ subgenomes of ${\mathcal {G}}$ and ${\mathcal {H}}$, the set ${\mathbf {T}}^{\mathbf {min}}_{{\mathcal {G}},{\mathcal {H}}}$ can be constructed in $O(n^3)$ time. We can also store which subgenomes belong to the set ${\mathbf {T}}^{\mathbf {min}}_{{\mathbb {S}}^{\mathbf {i}},{\mathbb {P}}^{\mathbf {i}}}$ in a suffix tree allowing the update of $\mathbf {T}^{\mathbf {min}}_{{\mathbb {S}}^{\mathbf {i}},{\mathbb {P}}^{\mathbf {i}}}$ in *O*(*n*) time. Additionally, we can store the known breakpoints in a binary search tree so it is possible to recover $\texttt {break}({\mathcal {X}})$ in $O(n\log n)$ time. The initialization of Algorithm 1 (lines 1 to 4) takes $O(n^3)$ time, the loop from lines 5 to 16 is repeated at most *O*(*n*) times, because there are at most 2*n* breakpoints, and each iteration takes at most $O(n \log n)$ time, since searching the breakpoint takes time $O(n \log n)$ and updating $\mathbf {T}^{\mathbf {min}}_{{\mathbb {S}}^{\mathbf {i}},{\mathbb {P}}^{\mathbf {i}}}$ takes linear time. Consequently, Algorithm 1 has time complexity $O(n^3)$.

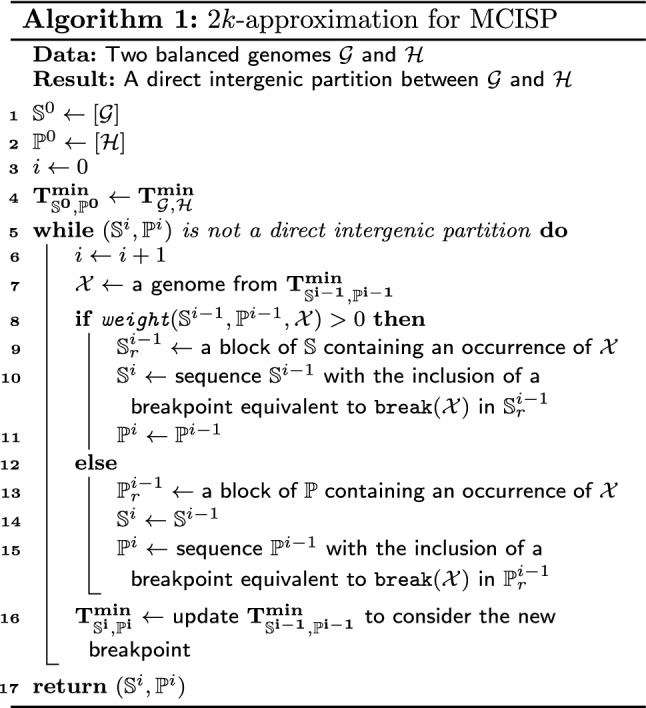



### Example 3

Execution of Algorithm 1 with genomes ${\mathcal {G}}= (S,\breve{S})$ and ${\mathcal {H}}= (P,\breve{P})$. In a genome ${\mathcal {X}}$, the intergenic region correspondent to $\texttt {break}({\mathcal {X}})$ is marked in bold.

$$\begin{aligned} S&~=~ [I\,\, B\,\, A\,\, B\,\, C\,\, B\,\, C\,\, F]~~~~~~ \breve{S} ~=~ [1\,\, 3\,\, 2\,\, 3\,\, 1\,\, 3\,\, 2]\\ P&~=~ [I\,\, B\,\, C\,\, A\,\, B\,\, B\,\, C\,\, F]~~~~~~ \breve{P} ~=~ [1\,\, 3\,\, 0\,\, 2\,\, 4\,\, 3\,\, 2]\\ {\mathbf {T}}^{\mathbf {min}}_{{\mathcal {G}},{\mathcal {H}}}&~=~ \{([B\,\, A], [{\mathbf {3}}]), ([A\,\, B\,\, C], [{{\mathbf {2}}\,\, 3}]), ([{C\,\, B}], [{{\mathbf {1}}}]),\\&~~~~~~~~(([{I\,\, B\,\, C}], [{{\mathbf {1}}\,\, 3}])[{C\,\, A}], [{{\mathbf {0}}}]), ([{B\,\, B}], [{{\mathbf {4}}}])\}\\ {\mathbb {S}}^0&~=~ [({[{I\,B\,A\,B\,C\,B\,C\,F}], [{1\,3\,2\,3\,1\,3\,2}]})]\\ {\mathbb {P}}^0&~=~ [({[{I\,B\,C\,A\,B\,B\,C\,F}], [{1\,3\,0\,2\,4\,3\,2}]})]\\ \mathbf {T}^{\mathbf {min}}_{{\mathbb {S}}^{\mathbf {0}},{\mathbb {P}}^{\mathbf {0}}}&~=~ {\mathbf {T}}^{\mathbf {min}}_{{\mathcal {G}},{\mathcal {H}}}\\ {\mathbb {S}}^1&~=~ [{([{I\, B}],[1])\quad ([{A\,B\,C\,B\,C\,F}], [{2\,3\,1\,3\,2}])}]\\ {\mathbb {P}}^1&~=~ {\mathbb {P}}^0\\ {\mathbf {T}}^{\mathbf {min}}_{{\mathbb {S}}^{\mathbf {1}},{\mathbb {P}}^{\mathbf {1}}}&~=~ \{([{A\,\, B\,\, C}], [{{\mathbf {2}}\,\, 3}]), ([C\,\, B], [{{\mathbf {1}}}]), ([{I\,\, B\,\, C}], [{{\mathbf {1}}\,\, 3}]),\\&~~~~~~~~([C\,\, A], [{{\mathbf {0}}}]), ([B\,\, B], [{{\mathbf {4}}}])\}\\ {\mathbb {S}}^2&~=~ [{([I\,B], [{1}])\quad ([{A}], [{~}])\quad ([{B\,C\,B\,C\,F}], {3\,1\,3\,2})}]\\ {\mathbb {P}}^2&~=~ {\mathbb {P}}^1\\ {\mathbf {T}}^{\mathbf {min}}_{{\mathbb {S}}^{\mathbf {2}},{\mathbb {P}}^{\mathbf {2}}}&~=~ \{([C\,\, B], [{{\mathbf {1}}}]), ([{I\,\, B\,\, C}], [{{\mathbf {1}}\,\, 3}]), ([{C\,\, A}], [{{\mathbf {0}}}]),\\&~~~~~~~~([B\,\, B], [{\mathbf {4}}]), ([A\,\, B], [{{\mathbf {2}}}])\}\\ {\mathbb {S}}^3&~=~ [{({[I\,B]}, [{1}])\quad ([{A}], [{~}])\quad ([{B\,C}], [{3}]),}\\&~~~~~~~{({[B\,C\,F}],[{3\,2]})}]\\ {\mathbb {P}}^3&~=~ {\mathbb {P}}^2\\ {\mathbf {T}}^{\mathbf {min}}_{{\mathbb {S}}^{\mathbf {3}},{\mathbb {P}}^{\mathbf {3}}}&~=~ \{([I\,\, B\,\, C], [{\mathbf {1}}\,\, 3]), ([C\,\, A], [{{\mathbf {0}}}]), ([B\,\, B], [{{\mathbf {4}}}]),\\&~~~~~~~~([A\,\, B], [{{\mathbf {2}}}])\}\\ {\mathbb {S}}^4&~=~ {\mathbb {S}}^3\\ {\mathbb {P}}^4&~=~ [{([I],[{~}])\quad ([{B\,C\,A\,B\,B\,C\,F}], {[3\,0\,2\,4\,3\,2]})}]\\ {\mathbf {T}}^{\mathbf {min}}_{{\mathbb {S}}^{\mathbf {4}},{\mathbb {P}}^{\mathbf {4}}}&~=~ \{([{C\,\, A}], [{{\mathbf {0}}}]), ([{B\,\, B}], [{{\mathbf {4}}}]), ([{A\,\, B}], [{{\mathbf {2}}}]),\\&~~~~~~~~([I\,\, B], [{{\mathbf {1}}}])\}\\ {\mathbb {S}}^5&~=~ {\mathbb {S}}^4\\ {\mathbb {P}}^5&~=~ [{([I], [{~}])\quad ({(B\,C)}, [{3}])\quad ([{A\,B\,B\,C\,F}], [{2\,4\,3\,2}])}]\\ {\mathbf {T}}^{\mathbf {min}}_{{\mathbb {S}}^{\mathbf {5}},{\mathbb {P}}^{\mathbf {5}}}&~=~ \{([B\,\, B], [{{\mathbf {4}}}]), ([A\,\, B], [{{\mathbf {2}}}]), ([{I\,\, B}], [{{\mathbf {1}}}])\}\\ {\mathbb {S}}^6&~=~ {\mathbb {S}}^5\\ {\mathbb {P}}^6&~=~ [{([{I}], [{~}])\quad ([{B\,C}], [{3}]), ([{A\,B}],[{2}])}\\&~~~~~~~{([{B\,C\,F}], [{3\,2}])}]\\ {\mathbf {T}}^{\mathbf {min}}_{{\mathbb {S}}^{\mathbf {6}},{\mathbb {P}}^{\mathbf {6}}}&~=~ \{([{A\,\, B}], [{{\mathbf {2}}}]), ([I\,\, B], [{{\mathbf {1}}}])\}\\ {\mathbb {S}}^7&~=~ {\mathbb {S}}^6\\ {\mathbb {P}}^7&~=~ [{([{I}],[{~}])\quad ([{B\,C}], [{3}])\quad ([{A}],[{~}])\quad ([{B}],[{~}])}\\&~~~~~~~{([{B\,C\,F}], [{3\,2}])}]\\ {\mathbf {T}}^{\mathbf {min}}_{{\mathbb {S}}^{\mathbf {7}},{\mathbb {P}}^{\mathbf {7}}}&~=~ \{([I\,\, B], [{{\mathbf {1}}}])\}\\ {\mathbb {S}}^8&~=~ [{([{I}], [{~}])\quad ([{B}], [{~}])\quad ([{A}], [{~}])\quad ([{B\,C}], [{3}])}\\&~~~~~~~{([{B\,C\,F}],[{3\, 2}])}]\\ {\mathbb {P}}^8&~=~ {\mathbb {P}}^7\\ {\mathbf {T}}^{\mathbf {min}}_{{\mathbb {S}}^{\mathbf {8}},{\mathbb {P}}^{\mathbf {8}}}&~=~ \{\} \end{aligned}$$

### Lemma 14

*Algorithm* 1 *produces a direct intergenic partition of two genomes* ${\mathcal {G}}= (S,\breve{S})$ *and* ${\mathcal {H}}= (P, \breve{P})$, *including at most* $2k|{\mathbf {T}}^{\mathbf {min}}_{{\mathcal {G}},{\mathcal {H}}}|$ *breakpoints, where* $k = occ(S)$.

### Proof

Initially, we show that the algorithm stops producing a direct intergenic partition, i.e., eventually $\texttt {weight}({\mathbb {S}}^{i},{\mathbb {P}}^{i},{\mathcal {X}}) = 0$. At every step we reduce the occurrence of at least one genome in ${\mathbb {S}}^{i}$ or in ${\mathbb {P}}^{i}$ and, while $\texttt {weight}({\mathbb {S}}^{i},{\mathbb {P}}^{i},{\mathcal {X}}) \ne 0$, there is an element in ${\mathbf {T}}^{\mathbf {min}}_{{\mathbb {S}}^{\mathbf {i}},{\mathbb {P}}^{\mathbf {i}}}$ where we can insert a breakpoint. As the number of occurrences of genomes in ${\mathbb {S}}^{i}$ and in ${\mathbb {P}}^{j}$ is finite, integer, non-negative, and always decreasing, eventually the algorithm stops with $\texttt {weight}({\mathbb {S}}^{i},{\mathbb {P}}^{i},{\mathcal {X}}) = 0$.

Now, we show that we include at most $2k|{\mathbf {T}}^{\mathbf {min}}_{{\mathcal {G}},{\mathcal {H}}}|$ breakpoints. Every breakpoint is included in an occurrence of a genome from ${\mathbf {T}}^{\mathbf {min}}_{{\mathcal {G}},{\mathcal {H}}}$ or is equivalent to an already included breakpoint. Consequently, every breakpoint is equivalent to $\texttt {break}({\mathcal {X}})$ for some ${\mathcal {X}}\in {\mathbf {T}}^{\mathbf {min}}_{{\mathcal {G}},{\mathcal {H}}}$. As there is a maximum of *k* copies for each gene in ${\mathcal {G}}$ and a maximum of *k* copies for each gene in ${\mathcal {H}}$, every breakpoint is equivalent to a maximum of $2k-1$ other breakpoints, so we include at most $2k|{\mathbf {T}}^{\mathbf {min}}_{{\mathcal {G}},{\mathcal {H}}}|$ breakpoints. $\square$

### Theorem 7

  *Algorithm* 1 *has an approximation factor of* 2*k* *for the MCISP problem between the genomes* ${\mathcal {G}}= (S,\breve{S})$ *and* ${\mathcal {H}}= (P, \breve{P})$, *where* $k = occ(S)$.

### Proof

Directly from lemmas 13 and 14. $\square$

### Corollary 1

*Algorithm* 1 *has an approximation factor of 2k for the MCSP problem between the string* *S* *and* *P*, *where* $k = occ(S)$.

### Proof

Using the same reduction presented in Theorem 2, but considering the optimization versions of the problems, we can apply Algorithm 1 to the MCSP problem and ensure the approximation factor 2*k*. $\square$

It is worth noting that we improve the previously known $\Theta (k)$ approximation of MCSP [20] from 4*k* to 2*k*.

### Corollary 2

*Algorithm* 1, *in combination with the algorithm described in Lemma* 9, *ensures an asymptotic approximation factor of* 6*k* *for the ITD problem between the genomes *$\mathcal{G} = (S,\breve{S})\ and \ \mathcal{H} = (P,\breve{P})$, *where* $k = occ(S)$.

### Proof

Directly from theorems 4 and 7. $\square$

## 2k-approximation for RMCISP

We can adapt Algorithm 1 to approximate the RMCISP problem. The main point of the adaptation is to use congruence of genomes instead of equality and substitute the relation ${\mathcal {X}}\subset {\mathcal {G}}$ with a new relation ${\mathcal {X}}\sqsubset {\mathcal {G}}$, such that ${\mathcal {X}}\sqsubset {\mathcal {G}}$ if ${\mathcal {X}}\subset {\mathcal {G}}$ or $rev({\mathcal {X}}) \subset {\mathcal {G}}$. Using this relation, the functions and sets from the previous section must be adapted:

- $\texttt {subgen}({\mathcal {G}},{\mathcal {X}})$ is now the number of subgenomes of ${\mathcal {G}}$ congruent to ${\mathcal {X}}$ (i.e., equal to ${\mathcal {X}}$ or to $rev({\mathcal {X}})$). Consequently, $\texttt {weight}$ considers now this new $\texttt {subgen}$ function.
- ${\mathbf {T}}_{{\mathcal {G}},{\mathcal {H}}}$ is now the set of all genomes ${\mathcal {X}}$, such that ${\mathcal {X}}\sqsubset {\mathcal {G}}$ or ${\mathcal {X}}\sqsubset {\mathcal {H}}$, and $\texttt {weight}({\mathcal {G}},{\mathcal {H}},{\mathcal {X}}) \ne 0$. Additionally, ${\mathbf {T}}^{\mathbf {min}}_{{\mathcal {G}},{\mathcal {H}}} = \{{\mathcal {X}}\in {\mathbf {T}}_{{\mathcal{G}},{\mathcal {H}}}| {\mathcal{Y}}/ \!\!\kern-7pt \sqsubset {\mathcal {X}}, \forall {\mathcal {Y}}\in \mathbf{T}_{{\mathcal {G}},{\mathcal {H}}, {\mathcal {Y}}\ne{\mathcal {X}}} \}$ (${\mathbf {T}}^{\mathbf {min}}_{{\mathbb {S}},{\mathbb {P}}}$ is adapted in a similar manner).

Some other adaptations must be made on Algorithm 1. Line 5 must check if $({\mathbb {S}},{\mathbb {P}})$ is a reverse intergenic partition instead of a direct intergenic partition. In lines 9 and 13, the block must contain an occurrence of ${\mathcal {X}}$ or $rev({\mathcal {X}})$, and the breakpoint in lines 10 and 14 must be congruent to $\texttt {break}({\mathcal {X}})$ instead of equivalent to $\texttt {break}({\mathcal {X}})$. Next, we show analogous results to the ones presented in the previous section.

### Lemma 15

  *Given two genomes* ${\mathcal {G}}= (S,\breve{S})$, ${\mathcal {H}}= (P,\breve{P})$, *and a pair* $({\mathbb {S}}, {\mathbb {P}})$ *of genome sequences, such that it satisfies conditions* 1 *and* 2 *of reverse intergenic partition, we have that* $({\mathbb {S}}, {\mathbb {P}})$ *satisfies condition* 3 *if and only if* $\texttt {weight}({\mathbb {S}},{\mathbb {P}},{\mathcal {X}}) = 0$ *for all genomes* ${\mathcal {X}}$, *such that* ${\mathcal {X}}\sqsubset {\mathcal {G}}$ or ${\mathcal {X}}\sqsubset {\mathcal {H}}$.

### Proof

First, we argue that if the third condition is satisfied then $\texttt {weight}({\mathbb {S}},{\mathbb {P}},{\mathcal {X}}) = 0$. Assuming the third condition is satisfied, we have a permutation $\phi$, from the numbers 1 to $|{\mathbb {S}}|$, such that ${\mathbb {P}}_i \cong {\mathbb {S}}_{\phi _i}$, $\forall ~{1 \le i \le |{\mathbb {S}}|}$.

Let ${\mathcal {X}}$ be a genome such that ${\mathcal {X}}\sqsubset {\mathcal {G}}$ or ${\mathcal {X}}\sqsubset {\mathcal {H}}$. In $\texttt {weight}({\mathbb {S}},{\mathbb {P}},{\mathcal {X}})$, we are only going to count an occurrence of ${\mathcal {X}}$ or $rev({\mathcal {X}})$ in ${\mathcal {G}}$ if it is a subgenome of some block of ${\mathbb {S}}$. Similarly, we are only going to count an occurrence of ${\mathcal {X}}$ or $rev({\mathcal {X}})$ in ${\mathcal {H}}$ if it is a subgenome of some block of ${\mathbb {P}}$.

Note that the counted occurrences of ${\mathcal {X}}$ or $rev({\mathcal {X}})$ in ${\mathcal {G}}$ are in a one-to-one correspondence with the counted occurrences in ${\mathcal {H}}$. More precisely, for a subgenome ${\mathbb {S}}_k^{i,j}$ of a block ${\mathbb {S}}_k$ such that ${\mathbb {S}}_k^{i,j} \cong {\mathcal {X}}$, there is a subgenome ${\mathbb {P}}_{\phi _k}^{i,j}$ of a block ${\mathbb {P}}_{\phi _k}$, such that ${\mathbb {P}}_{\phi _k}^{i,j} \cong {\mathcal {X}}$. Conversely, for a subgenome ${\mathbb {P}}_{\phi _k}^{i,j}$ of a block ${\mathbb {P}}_{\phi _k}$, such that ${\mathbb {P}}_{\phi _k}^{i,j} \cong {\mathcal {X}}$ there is a subgenome ${\mathbb {S}}_{k}^{i,j}$ of a block ${\mathbb {S}}_{k}$, such that ${\mathbb {S}}_{k}^{i,j} \cong {\mathcal {X}}$. Consequently, $\texttt {weight}({\mathbb {S}},{\mathbb {P}},{\mathcal {X}}) = 0$ for every genome ${\mathcal {X}}$, such that ${\mathcal {X}}\sqsubset {\mathcal {G}}$ or ${\mathcal {X}}\sqsubset {\mathcal {H}}$.

Now we prove that if $\texttt {weight}({\mathbb {S}},{\mathbb {P}},{\mathcal {X}}) = 0$ then the third condition is satisfied. By contradiction let us assume that there is no one-to-one correspondence between blocks of ${\mathbb {S}}$ and blocks of ${\mathbb {P}}$.

The impossibility of a correspondence may happen by four reasons: (*i*) there is a block in ${\mathbb {S}}$ that is not congruent to any block of ${\mathbb {P}}$; (*ii*) there is a genome ${\mathcal {X}}$ congruent to *r* blocks of ${\mathbb {S}}$, but it is congruent to $\ell < r$ blocks of ${\mathbb {P}}$; (*iii*) there is a block in ${\mathbb {P}}$ not congruent to any block of ${\mathbb {S}}$; (*iv*) there is a genome ${\mathcal {X}}$ congruent to *r* blocks of ${\mathbb {P}}$, but it is congruent to $\ell < r$ blocks of ${\mathbb {S}}$. Without loss of generality, we consider only the first two cases.

In case (*i*), assume that ${\mathbb {S}}_j$ is the biggest block of ${\mathbb {S}}$ not congruent to any block of ${\mathbb {P}}$. As $\texttt {weight}({\mathbb {S}},{\mathbb {P}},{\mathbb {S}}_j) = 0$, we have $\sum _{i = 1}^{|{\mathbb {S}}|} \texttt {subgen}({\mathbb {S}}_i,{\mathbb {S}}_j) = \sum _{i = 1}^{|{\mathbb {P}}|} \texttt {subgen}({\mathbb {P}}_i,{\mathbb {S}}_j)$. Consequently, ${\mathbb {P}}$ must have a copy of ${\mathbb {S}}_j$ or $rev({\mathbb {S}}_j)$ in one of its blocks. Let ${\mathbb {P}}_s$ be a block with such copy, i.e., ${\mathbb {S}}_j \sqsubset {\mathbb {P}}_s$. If ${\mathbb {P}}_s \not \cong {\mathbb {S}}_j$, then ${\mathbb {S}}$ must have a copy of ${\mathbb {P}}_s$ or $rev({\mathbb {P}}_s$), because $\texttt {weight}({\mathbb {S}},{\mathbb {P}},{\mathbb {P}}_s) = 0$. This means that ${\mathbb {S}}$ has at least two copies of ${\mathbb {S}}_j$ or $rev({\mathbb {S}}_j)$ and we must have another copy of ${\mathbb {S}}_j$ or $rev({\mathbb {S}}_j)$ in ${\mathbb {P}}$. Following that argument, eventually ${\mathbb {P}}$ must have a block equal to ${\mathbb {S}}_j$ or $rev({\mathbb {S}}_j)$, contradicting the assumption of case (*i*).

In case (*ii*) we can establish a correspondence between the $\ell$ blocks of ${\mathbb {P}}$ and some of the *r* blocks of ${\mathbb {S}}$. We have at least one block of ${\mathbb {S}}$ without a correspondent in ${\mathbb {P}}$. If we ignore the blocks with correspondences when calculating the weights, the same argument of case (*i*) leads to a contradiction. $\square$

### Lemma 16

*In order to construct a reverse intergenic partition* $({\mathbb {S}}, {\mathbb {P}})$ *of two genomes* ${\mathcal {G}}$ *and* ${\mathcal {H}}$, *we must include a breakpoint in at least one copy of every element* ${\mathcal {X}}\in {\mathbf {T}}^{\mathbf {min}}_{{\mathcal {G}},{\mathcal {H}}}$.

### Proof

For a genome ${\mathcal {X}}\in {\mathbf {T}}_{{\mathcal {G}},{\mathcal {H}}}$, let $k = \texttt {weight}({\mathcal {G}},{\mathcal {H}},{\mathcal {X}})$. To ensure that $\texttt {weight}({\mathbb {S}},{\mathbb {P}},{\mathcal {X}}) = 0$, if $k > 0$, then we must include breakpoints in at least *k* copies of ${\mathcal {X}}$ or $rev({\mathcal {X}})$ in ${\mathcal {G}}$, otherwise, if $k < 0$, we must include breakpoints in at least $-k$ copies of ${\mathcal {X}}$ or $rev({\mathcal {X}})$ in ${\mathcal {H}}$. As $\texttt {weight}({\mathcal {G}},{\mathcal {H}},{\mathcal {X}}) \ne 0$, we must include at least one breakpoint in ${\mathcal {G}}$ or in ${\mathcal {H}}$, and the lemma follows. $\square$

### Lemma 17

*The adaptation of Algorithm* 1 *produces a direct intergenic partition of two genomes* ${\mathcal {G}}= (S,\breve{S})$ *and* ${\mathcal {H}}= (P, \breve{P})$, *including at most* $2k|{\mathbf {T}}^{\mathbf {min}}_{{\mathcal {G}},{\mathcal {H}}}|$ *breakpoints, where* $k = occ(S)$.

### Proof

We know the algorithm stops producing a reverse intergenic partition for the same reason stated in Lemma 14. Additionally, every breakpoint is included in an occurrence of a genome from ${\mathbf {T}}^{\mathbf {min}}_{{\mathcal {G}},{\mathcal {H}}}$ or is congruent to an already included breakpoint. Consequently, every breakpoint is congruent to $\texttt {break}({\mathcal {X}})$ for some ${\mathcal {X}}\in {\mathbf {T}}^{\mathbf {min}}_{{\mathcal {G}},{\mathcal {H}}}$. As there is a maximum of *k* copies for each gene in ${\mathcal {G}}$ and a maximum of *k* copies for each gene in ${\mathcal {H}}$, every breakpoint is congruent to a maximum of $2k-1$ other breakpoints, so we include at most $2k|{\mathbf {T}}^{\mathbf {min}}_{{\mathcal {G}},{\mathcal {H}}}|$ breakpoints. $\square$

### Theorem 8

*The adaptation of Algorithm* 1 *has an approximation factor of* 2*k* *for the RMCISP problem between the genomes* ${\mathcal {G}}= (S,\breve{S})$ *and* ${\mathcal {H}}= (P, \breve{P})$, *where* $k = occ(S)$.

### Proof

Directly from lemmas 16 and 17. $\square$

### Corollary 3

*The adaptation of Algorithm* 1 *has an approximation factor of* 2*k* *for the RMCSP problem between the string* *S* *and* *P*, *where* $k = occ(S)$.

### Proof

Applying a reduction, as in Corollary 1, we can apply the adaptation of Algorithm 1 to the RMCSP problem and ensure the approximation factor 2*k*. $\square$

It is worth noting that we improve the previously known $\Theta (k)$ approximation of RMCSP [20] from 8*k* to 2*k*.

### Corollary 4

*The adaptation of Algorithm* 1 *combined with the algorithm described by Brito* et al. [24] *for the Sorting Permutations by Intergenic Reversals problem ensures an approximation factor of* 8*k* *for the IRD problem between the strings* *S* *and* *P*, *where* $k = occ(S)$.

### Proof

Directly from theorems 5 and 8. $\square$

### Corollary 5

*The adaptation of Algorithm* 1 *combined with the algorithm described by Brito* et al. [24] *for the Sorting Permutations by Intergenic Reversals and Transpositions problem ensures an approximation factor of* 9*k* *for the IRTD problem between the genomes* $\mathcal{G} = (S,\breve{S})\ and \ \mathcal{H} = (P,\breve{P})$, *where* $k = occ(S)$.

### Proof

Directly from theorems 6 and 8. $\square$

## Experimental results

This section presents the results of our algorithms applied in databases of simulated genomes. Our partition algorithm was implemented in Haskell and the experiments were conducted on a PC equipped with a 2.3GHz Intel® Xeon® CPU E5-2470 v2, with 40 cores and 32 GB of RAM, running Ubuntu 18.04.2. We constructed one database for each rearrangement model: TRANS for intergenic transpositions, REV for intergenic reversals, and REVTRANS for intergenic reversals and transpositions. Each database has 40 sets of 100 genome pairs, and each set is defined by the size *m* of its correspondent alphabet and a number *o* of applied operations. Each pair of genomes was constructed as follows:

1. For the source genome ${\mathcal {G}}= (S,\breve{S})$, we constructed the string *S* by selecting 100 characters from a uniform distribution of *m* characters (correspondent to an alphabet $\Sigma$, such that $\Sigma _S \subset \Sigma$), each character could be selected more than once. Afterwards, we constructed the list $\breve{S}$ by randomly choosing each intergenic region from integers in the interval [0, 100], each integer had the same probability of being chosen.
2. For the target genome ${\mathcal {H}}= (P,\breve{P})$, we apply *o* operations in *S*. The type of operation depends on the database. In the TRANS database, we applied *o* intergenic transpositions $\tau ^{(i,j,k)}_{(x,y,z)}$, where the values of *i*, *j*, *k*, *x*, *y*, and *z* were randomly chosen. In the REV database, we applied *o* intergenic reversals $\rho ^{(i,j)}_{(x,y)}$, where the values *i*, *j*, *x*, and *y* were randomly chosen. In the REVTRANS database we applied $\left\lfloor \frac{o}{2} \right\rfloor$ intergenic reversals and $\left\lceil \frac{o}{2} \right\rceil$ intergenic transpositions. These operations were aplied in a random order and the parameters of each one were randomly chosen.
3. We performed the extension process by adding two extra characters in the extremities of the source and target genomes to ensure that they are co-tailed. Note that both genomes have a final size of 102.

In these tests, for each pair of genomes from the TRANS database, we computed the direct intergenic partition from our algorithm, and for each pair of genomes from REV and TRANSREV databases, we computed the reverse intergenic partition from our algorithm. Afterwards, we produced 100 orthologous assignments capable of inducing each partition. We ensured that each possible assignment had the same probability of being chosen.

For each assignment, we computed the distance between the genomes using the assignment. The distances are computed by a different algorithm for each database: for the TRANS database, we used the algorithm described in Lemma 9 (implemented in C++); for the REV and REVTRANS databases, we used the algorithms for reversals and reversals and transpositions from Brito et al. [24] (implemented in Python), respectively.

To compare with the distances that do not consider the partitions, we also produced, for each genome pair, 100 assignments that do not take into account the partitions. We computed the distances for each of these assignments as well.

Tables 2, 3, and 4 show the distances for the TRANS, REV, and REVTRANS databases, respectively. Each line corresponds to a set of 100 genome pairs; the first two columns indicate, respectively, the number of operations and the size of the alphabet used to generated the set. The following seven columns present the results considering the partitions. For each genome pair, we consider the minimum and average distance from all 100 assignments. For each set, we report the minimum (Min.), average (Avg.), and maximum (Max.) for those two values. We also report the average time, in seconds, necessary to produce the partition and compute the 100 distances. The last seven columns present the same values for the distances that do not consider the partitions. In that case, the time reported refers only to calculating the distances.

**Table 2:** Distances for the ITD problem with and without the use of our partition algorithm

OP	${|\Sigma |}$	With partition							Without partition						
		Minimum distance			Average distance			Time	Minimum distance			Average distance			Time
		Min.	Avg.	Max.	Min.	Avg.	Max.		Min.	Avg.	Max.	Min.	Avg.	Max.	
25	10	42	47.83	57	43.64	49.45	58.80	0.22	94	96.91	98	99.93	100.19	100.52	0.02
25	20	38	48.07	61	39.85	49.61	61.79	0.22	88	93.42	96	97.99	98.63	99.41	0.01
25	30	39	47.85	55	40.71	49.27	56.62	0.22	84	88.89	92	94.72	96.27	97.49	0.02
25	40	39	48.53	57	40.85	49.91	58.67	0.22	77	84.97	88	89.56	93.60	95.62	0.01
25	50	41	48.38	55	42.54	49.59	56.87	0.22	69	81.20	87	86.35	90.66	93.87	0.01
25	60	40	48.56	57	40.68	49.69	58.79	0.23	70	77.84	84	81.71	88.00	93.21	0.01
25	70	40	47.94	54	40.42	48.85	55.96	0.22	68	74.88	84	80.36	85.60	91.40	0.01
25	80	40	48.29	56	40.00	49.20	57.03	0.23	62	71.84	78	77.62	83.26	88.35	0.01
25	90	39	48.53	55	39.57	49.36	56.89	0.22	61	70.86	81	74.31	81.82	88.77	0.01
25	100	41	48.85	56	41.25	49.61	56.80	0.23	58	68.29	77	71.95	79.51	86.20	0.01
50	10	64	71.52	80	65.79	73.08	80.86	0.33	96	98.03	99	100.20	100.46	100.80	0.01
50	20	63	72.17	80	65.62	73.68	81.79	0.33	92	95.51	98	98.71	99.57	100.30	0.02
50	30	66	72.38	82	67.76	73.86	83.79	0.32	90	93.19	96	96.83	98.40	99.75	0.01
50	40	62	71.98	82	63.84	73.55	82.87	0.33	85	91.14	95	93.56	96.89	99.07	0.02
50	50	62	72.25	80	63.70	73.74	80.97	0.33	84	89.44	94	92.76	95.60	98.03	0.01
50	60	60	71.51	81	61.97	73.01	82.74	0.33	82	87.18	92	89.80	94.03	97.91	0.01
50	70	64	71.96	80	65.64	73.56	81.80	0.33	79	85.89	90	87.46	92.80	95.79	0.01
50	80	65	72.04	83	65.81	73.66	84.78	0.33	78	84.64	91	87.05	91.79	96.90	0.01
50	90	62	71.64	80	62.70	73.14	80.91	0.33	76	83.25	90	84.45	90.39	95.29	0.01
50	100	65	72.04	82	65.90	73.52	83.85	0.34	75	82.74	89	83.25	89.98	94.72	0.01
75	10	76	84.41	92	77.53	86.00	93.90	0.38	96	98.26	100	100.23	100.58	100.82	0.02
75	20	76	84.71	91	77.65	86.28	91.91	0.38	94	96.95	99	99.50	100.15	100.54	0.01
75	30	76	84.36	92	76.83	85.91	92.88	0.38	92	95.52	98	97.47	99.39	100.43	0.01
75	40	79	84.87	92	80.75	86.43	93.70	0.39	91	94.51	97	97.46	98.76	100.09	0.02
75	50	77	85.04	95	78.83	86.67	95.83	0.38	90	93.66	98	94.47	98.02	100.16	0.02
75	60	76	84.01	91	77.66	85.55	92.80	0.38	89	92.21	96	94.24	97.07	99.44	0.01
75	70	79	84.99	92	80.71	86.48	93.80	0.38	86	92.01	95	90.84	96.64	98.65	0.01
75	80	77	85.06	93	78.92	86.63	94.82	0.39	86	91.26	96	92.84	95.90	98.98	0.01
75	90	74	85.41	93	76.85	86.93	94.78	0.40	85	90.93	97	90.58	95.78	99.25	0.01
75	100	74	84.52	94	74.70	86.12	94.78	0.39	81	90.00	96	89.03	95.05	98.69	0.01
100	10	82	90.80	96	83.72	92.40	96.86	0.41	97	98.68	100	100.43	100.67	100.94	0.02
100	20	83	91.23	97	84.71	92.87	98.91	0.41	95	97.87	99	99.57	100.41	100.83	0.02
100	30	86	91.55	97	87.74	93.15	98.84	0.41	95	97.33	99	98.93	100.10	100.72	0.02
100	40	86	91.72	98	87.81	93.25	98.80	0.42	94	96.58	99	98.53	99.72	100.60	0.02
100	50	84	91.17	98	85.83	92.71	99.77	0.42	91	95.73	99	96.15	99.13	100.72	0.02
100	60	85	91.49	98	86.77	92.96	99.72	0.42	90	95.18	99	95.77	98.74	100.24	0.02
100	70	83	91.54	98	84.67	93.07	99.76	0.42	86	95.05	99	91.77	98.41	100.60	0.01
100	80	84	91.20	99	85.67	92.81	99.77	0.42	90	94.40	99	95.22	98.01	100.50	0.01
100	90	87	91.49	96	87.56	93.10	97.81	0.43	91	94.35	98	95.19	97.87	100.10	0.01
100	100	86	91.71	98	87.85	93.21	98.85	0.43	91	94.16	98	94.98	97.65	100.33	0.01

**Table 3:** Distances for the IRD problem with and without the use of our partition algorithm

OP	${|\Sigma |}$	With partition							Without Partition						
		Minimum Distance			Average Distance			Time	Minimum Distance			Average Distance			Time
		Min.	Avg.	Max.	Min.	Avg.	Max.		Min.	Avg.	Max.	Min.	Avg.	Max.	
25	10	27	33.38	41	30.65	37.20	47.07	5.35	98	100.44	102	105.15	105.84	106.82	14.43
25	20	27	32.64	38	29.44	35.36	41.36	5.11	93	98.04	101	102.56	104.56	105.80	14.21
25	30	28	32.55	38	29.25	34.90	40.69	4.99	88	95.02	99	99.35	102.59	104.50	13.83
25	40	28	32.91	39	28.00	34.65	40.48	4.98	84	92.17	98	95.01	100.47	104.07	13.71
25	50	28	32.62	38	28.00	34.13	39.42	4.96	80	89.46	96	92.96	98.65	102.58	13.26
25	60	27	32.61	39	27.00	33.85	40.81	4.94	78	87.57	98	91.02	97.03	104.22	12.99
25	70	28	32.35	39	28.00	33.64	40.90	4.93	73	85.74	94	87.39	95.04	101.30	12.71
25	80	27	32.69	38	28.04	33.58	39.50	4.97	72	83.86	95	84.09	93.49	101.54	12.40
25	90	27	32.79	38	27.00	33.55	40.39	4.94	72	82.31	92	82.03	91.70	99.34	12.03
25	100	28	32.78	38	28.00	33.48	39.24	4.92	66	81.82	95	80.16	91.52	101.39	12.04
50	10	54	60.94	68	56.67	64.24	71.66	9.38	98	100.83	103	105.48	106.04	106.86	14.48
50	20	52	59.96	68	55.80	63.24	72.77	9.29	97	99.30	101	103.80	105.11	106.16	14.37
50	30	51	59.91	70	53.45	63.03	73.07	9.22	92	97.56	100	101.59	104.05	106.35	14.19
50	40	51	59.86	68	53.22	62.93	71.84	9.28	90	95.72	100	99.79	102.76	104.98	13.93
50	50	50	59.70	67	51.83	62.82	71.34	9.08	89	94.04	100	97.50	101.47	104.42	13.73
50	60	54	59.69	67	55.81	62.55	69.59	9.13	84	92.74	99	93.63	100.16	105.08	13.44
50	70	52	59.48	65	54.02	62.42	69.31	9.02	84	91.46	98	92.73	99.12	103.50	13.34
50	80	51	59.30	68	53.04	62.05	71.22	8.91	83	90.18	97	91.74	97.89	103.89	13.12
50	90	53	59.65	66	55.03	62.23	68.75	9.01	79	89.12	95	89.68	96.90	102.52	12.99
50	100	51	58.68	65	53.30	61.24	68.84	8.96	77	88.71	98	86.47	96.52	102.28	13.00
75	10	67	75.02	81	69.82	78.41	83.89	11.51	99	101.33	103	105.40	106.17	106.67	14.52
75	20	67	75.19	83	70.95	78.60	86.92	11.43	97	99.94	102	104.55	105.55	106.39	14.35
75	30	67	74.70	83	70.08	78.23	86.72	11.56	94	98.79	102	103.79	104.89	106.20	14.33
75	40	68	75.63	82	70.68	79.10	85.96	11.53	93	98.20	101	101.55	104.17	105.73	14.23
75	50	68	74.52	82	70.73	77.93	84.80	11.37	89	96.76	100	99.61	103.13	105.25	14.08
75	60	67	75.07	84	71.77	78.33	88.08	11.44	90	95.81	100	98.14	102.43	105.60	13.87
75	70	63	75.03	85	65.25	78.32	88.19	11.44	86	95.35	100	97.33	101.91	105.08	14.00
75	80	67	74.70	81	69.33	77.70	84.76	11.31	86	94.74	99	95.42	101.16	105.35	13.65
75	90	68	74.97	82	70.67	77.94	85.61	11.50	87	94.00	99	96.41	100.67	106.91	13.73
75	100	65	74.49	82	69.30	77.59	85.82	11.18	84	93.34	100	92.97	99.93	103.94	13.48
100	10	77	83.54	91	80.65	87.27	95.42	13.12	98	101.23	103	105.77	106.25	106.79	14.56
100	20	77	83.34	90	80.43	87.14	93.44	12.89	97	100.65	103	104.04	105.80	106.69	14.45
100	30	75	83.73	91	78.56	87.41	94.77	13.17	97	99.67	102	102.77	105.30	106.59	14.39
100	40	77	83.78	92	79.85	87.43	96.10	12.89	94	98.77	101	100.29	104.46	106.72	14.20
100	50	76	83.76	91	80.52	87.36	95.46	13.02	94	98.42	102	100.86	104.17	106.55	14.25
100	60	77	84.05	91	79.73	87.58	95.06	13.12	93	97.50	101	99.96	103.51	105.93	14.05
100	70	77	84.40	92	79.93	87.91	95.21	12.97	93	97.38	101	99.26	103.16	106.34	14.03
100	80	76	83.95	92	79.82	87.46	95.58	12.95	91	96.94	101	97.99	102.77	105.72	13.91
100	90	77	84.10	93	80.15	87.50	97.68	12.84	93	96.56	101	99.07	102.37	106.41	13.80
100	100	74	84.09	90	76.45	87.45	93.49	13.02	89	96.41	101	96.20	102.10	105.84	13.87

**Table 4:** Distances for the IRTD problem with and without the use of our partition algorithm

OP	${|\Sigma |}$	With partition							Without partition						
		Minimum distance			Average distance			Time	Minimum distance			Average distance			Time
		Min.	Avg.	Max.	Min.	Avg.	Max.		Min.	Avg.	Max.	Min.	Avg.	Max.	
25	10	34	42.01	52	35.07	43.70	52.51	6.86	95	97.08	98	99.61	100.12	100.50	12.59
25	20	32	41.89	50	32.53	43.38	51.06	6.74	91	94.79	98	97.78	99.10	99.84	12.53
25	30	34	41.79	48	34.74	43.18	49.83	6.76	85	92.07	96	95.73	97.69	99.68	12.39
25	40	34	41.39	47	35.55	42.60	48.38	6.78	81	89.38	94	91.59	95.91	98.63	12.31
25	50	35	41.97	48	35.00	43.03	50.70	6.68	79	87.64	94	89.88	94.51	98.58	12.28
25	60	37	41.65	48	37.00	42.60	48.27	6.64	77	85.67	93	87.68	93.16	97.16	12.07
25	70	34	41.73	49	34.98	42.68	49.44	6.73	73	82.57	92	85.13	90.79	97.24	11.84
25	80	37	42.26	49	37.54	43.05	50.05	6.83	69	81.44	89	79.81	89.54	95.94	11.73
25	90	35	41.57	48	35.76	42.34	48.57	6.62	71	79.51	89	79.79	87.65	95.32	11.54
25	100	35	41.84	48	36.00	42.57	49.22	6.71	66	79.31	89	77.86	87.45	94.45	11.43
50	10	59	66.72	75	60.61	67.91	76.42	10.13	95	97.64	99	99.91	100.26	100.50	12.59
50	20	57	66.93	74	58.60	68.14	74.67	10.03	94	96.08	98	98.93	99.63	100.39	12.50
50	30	60	67.11	76	61.56	68.25	77.61	10.10	90	94.31	97	97.01	98.73	99.85	12.45
50	40	56	66.21	73	56.47	67.35	74.62	9.97	86	92.72	96	95.12	97.72	99.91	12.36
50	50	59	66.79	74	60.41	67.96	75.60	10.04	87	91.76	96	94.44	96.98	99.49	12.29
50	60	57	66.32	75	57.74	67.39	76.48	10.12	81	89.80	94	92.17	95.72	98.76	12.33
50	70	53	66.66	75	53.77	67.86	76.61	9.88	83	89.43	95	88.72	94.97	98.51	12.16
50	80	59	66.69	72	59.68	67.74	73.50	9.84	77	88.68	95	89.37	94.34	98.27	12.25
50	90	53	66.24	75	54.56	67.30	76.58	10.09	80	87.89	94	87.04	93.64	97.91	12.16
50	100	59	66.36	76	59.44	67.33	76.59	9.85	80	87.03	94	87.12	92.81	96.63	12.06
75	10	71	79.44	86	72.64	80.61	86.68	11.86	96	97.82	99	100.07	100.35	100.61	12.62
75	20	74	79.92	88	74.53	81.05	88.75	11.84	95	96.88	99	98.85	99.94	100.45	12.55
75	30	71	79.72	87	72.55	80.99	88.57	11.94	92	95.69	98	97.62	99.37	100.19	12.50
75	40	69	79.47	88	70.51	80.64	89.64	11.84	90	94.72	97	96.37	98.67	99.91	12.43
75	50	70	80.38	88	70.72	81.49	89.60	12.00	86	93.89	97	95.04	98.09	100.15	12.44
75	60	72	80.04	89	72.59	81.13	89.67	11.88	88	93.55	97	94.59	97.71	99.60	12.43
75	70	72	80.72	89	72.52	81.78	89.68	12.00	88	92.89	97	93.54	97.06	99.82	12.29
75	80	71	79.91	89	72.68	81.05	89.76	11.86	87	91.95	97	93.33	96.27	99.12	12.44
75	90	73	79.81	88	73.65	80.84	88.63	11.98	88	91.74	96	92.58	96.00	99.04	12.37
75	100	73	80.26	89	73.63	81.32	90.53	11.84	85	91.36	96	90.71	95.55	99.20	12.18
100	10	80	86.23	95	80.56	87.44	96.71	13.20	97	98.18	99	100.13	100.42	100.72	12.62
100	20	81	86.61	94	81.70	87.78	94.59	13.26	95	97.20	99	99.28	100.09	100.56	12.54
100	30	79	87.14	93	80.63	88.39	93.81	13.29	94	96.59	98	98.59	99.73	100.30	12.59
100	40	80	87.15	92	81.57	88.31	93.67	13.19	93	95.84	98	97.28	99.29	100.17	12.50
100	50	79	86.92	93	80.53	88.02	94.68	13.18	91	95.26	97	96.09	98.81	100.18	12.47
100	60	79	87.14	93	80.47	88.27	94.56	13.15	90	94.89	98	96.08	98.52	100.15	12.42
100	70	81	87.61	92	82.67	88.71	93.64	13.21	90	94.70	98	94.98	98.21	100.08	12.37
100	80	78	87.98	96	79.69	89.09	96.57	13.47	91	94.55	97	95.46	98.01	99.97	12.50
100	90	79	87.04	93	80.66	88.22	93.65	13.16	89	94.14	97	93.84	97.64	99.72	12.32
100	100	78	87.46	93	80.53	88.50	94.68	13.13	88	93.65	98	92.55	97.19	100.04	12.24

**Fig. 8:**
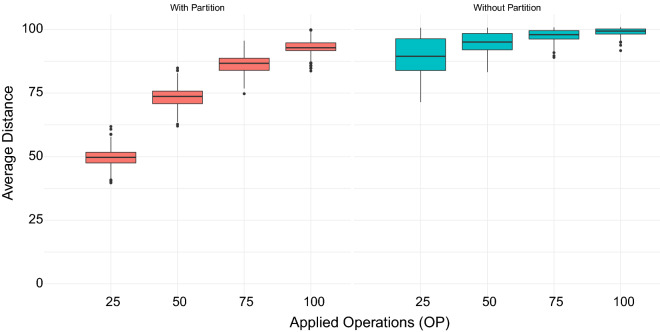
Average distances for the ITD problem with and without the use of our partition algorithm

**Fig. 9:**
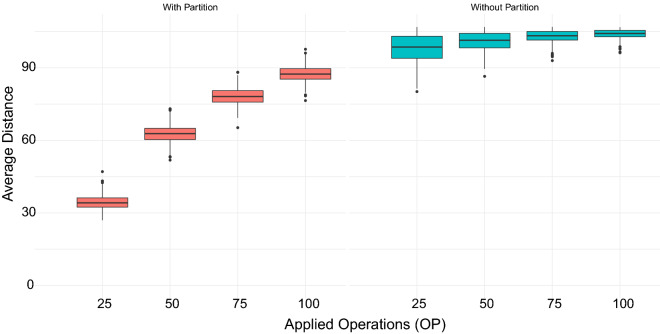
Average distances for the IRD problem with and without the use of our partition algorithm

**Fig. 10:**
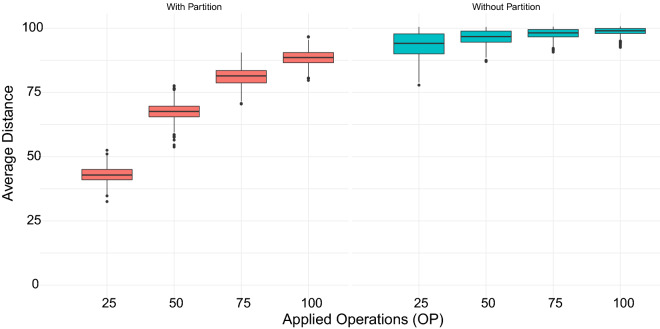
Average distances for the IRTD problem with and without the use of our partition algorithm

Figures 8, 9, and 10 show box plots with the average distances for the TRANS, REV, and REVTRANS databases, respectively.

From Table 2 and Fig. 8, we see that in the TRANS database the distances considering the partitions are lower than the distances that do not take the partitions into account. For sets generated with 25 transpositions, the minimum distances without partition are, on average, at least $39\%$ higher than the minimum distances with partition. For the average distance, the difference is at least $60\%$ on average. The difference between the distances decreases as the number of operations or the size of the alphabet increases. For sets generated with 100 transpositions and alphabet of size 10, the minimum and average distances without partition are on average $8\%$ higher than the minimum or average distances with partition. For sets generated with 100 transpositions and alphabet of size 100, the minimum distances without partition are on average $3\%$ higher than the minimum distances with partition. For the average distance, the difference is $5\%$ on average. It is worth mentioning that with 100 operations we have an extreme case, where each origin genome is considerably shuffled to produce the corresponding target genome of the pair. It is also interesting that with smaller alphabets, when the number of replicas increases, the advantage of using the partitions also increases. Looking at the running times, we see that, for the transposition model, we must pay a small cost to produce better distances using the partitions.

From Table 3 and Fig. 9, we see that in the REV database the distances considering the partitions are still lower than the distances that do not take the partitions into account, and the differences between distances are higher for this database. For sets generated with 25 reversals, the minimum distances without partition are, on average, at least $149\%$ higher than the minimum distances with partition. For the average distance, the difference is at least $173\%$ on average. Again, the difference between the distances decreases as the number of operations or the size of the alphabet increases, however, even in sets generated with 100 reversals and alphabet of size 100, the minimum distances with partition are on average $14\%$ higher than the minimum distances with partition. For the average distance, the difference is $16\%$ on average. In the REV database, we see that the running time considering the partition was lower than the running time without the partition. This happened because the 100 runs of the distance algorithm were slower than the partition algorithm, and using assignments that consider the partition tends to reduce the running time of the distance algorithm as the number of breakpoints tends to be smaller than the number of breakpoints considering a random assignment.

From Table 4 and Fig. 10, we see that in the REVTRANS database the distances considering the partitions are still lower than the distances that do not take the partitions into account. The differences were higher than those from the TRANS database, but smaller than those from the REV database. For sets generated with 25 operations, the minimum distances without partition are, on average, at least $90\%$ higher than the minimum distances with partition. For the average distance, the difference is at least $105\%$ on average. Again, the difference between the distances decreases as the number of operations or the size of the alphabet increases. In sets generated with 100 operations and alphabet of size 100, the minimum distances with partition are on average $7\%$ higher than the minimum distances with partition. For the average distance, the difference is $10\%$ on average. For the set generated with at most 75 operations, the running time considering the partition was lower than the running time without the partition.

Considering all results, we see that the partitions improve the distances and the improvement is higher for smaller alphabets or closer genomes (genomes that can be turned into one another with fewer operations). We can also see that with partitions, we have either a small cost in the running time, when the distance algorithm takes less time than the partition algorithm, or a large gain in running time, when the distance algorithm takes more time than the partition algorithm.

## Conclusion

We defined the intergenic transposition distance (ITD), the intergenic reversal distance (IRD), the intergenic reversal and transposition distance (IRTD), the minimum common intergenic string partition (MCISP), and the reverse minimum common intergenic string partition (RMCISP) problems. Next, we described a relation between the partition and distance problems and a $\Theta (k)$-approximation for the MCISP and RMCISP problems ensuring a $\Theta (k)$-approximation for the ITD, IRD, and IRTD problems. Our algorithm for the MCISP and RMCISP problems may also be applied to the MCSP and RMCSP problems, which do not consider intergenic regions, improving a previously known approximation. We also performed practical tests on simulated genomes, showing that the distances calculated considering the partitions were lower than the distances calculated without taking partitions into account.

As future works, one can extend our approach by considering the orientation of the genes. Additionally, one possible approach to overcome the balanced genome restriction is to consider non-conservative events, such as insertion and deletion, similarly to the work of Alexandrino et al. [30] with the Intergenic Reversal Distance without gene repetition.

## Data Availability

The algorithms and datasets generated during the current study are available in the following public repository: https://github.com/compbiogroup/Approximation-Algorithm-for-Rearrangement-Distances-Considering-Repeated-Genes-and-Intergenic-Region.
